# Antiparasitic and Antifungal Activities of Cetyl-Maritima, a New *N*-Cetyl-Modified Maritima Derivative

**DOI:** 10.3390/antibiotics14030321

**Published:** 2025-03-19

**Authors:** Ibrahim S. Al Nasr, Jingyi Ma, Tariq A. Khan, Waleed S. Koko, Imen Ben Abdelmalek, Rainer Schobert, Wendy van de Sande, Bernhard Biersack

**Affiliations:** 1 Department of Biology, College of Science, Qassim University, Buraydah 51452, Saudi Arabia; insar@qu.edu.sa (I.S.A.N.); wasyko2002@yahoo.com (W.S.K.); mm.abdulmalek@qu.edu.sa (I.B.A.); 2 Department of Medical Microbiology and Infectious Diseases, Erasmus MC, University Medical Center Rotterdam, Dr. Molewaterplein 40, 3015 GD Rotterdam, The Netherlands; m.jingyi@erasmusmc.nl (J.M.); w.vandesande@erasmusmc.nl (W.v.d.S.); 3 Department of Basic Health Sciences, College of Applied Medical Sciences, Qassim University, Buraydah 51452, Saudi Arabia; sirtariqayub@gmail.com; 4 Organic Chemistry Laboratory, University Bayreuth, Universitätsstrasse 30, 95440 Bayreuth, Germany; rainer.schobert@uni-bayreuth.de

**Keywords:** fragrances, Maritima, neglected tropical diseases, antiparasitic drugs, antifungal agents, mycetoma, *Madurella mycetomatis*, toxoplasmosis, leishmaniasis, *Leishmania major*

## Abstract

**Background/Objectives:** New drugs are urgently needed for the treatment of neglected tropical diseases including leishmaniasis and eumycetoma, as well as globally occurring parasitic diseases such as toxoplasmosis. Fragrances, both natural and synthetic, were shown to be a rich source for the development of new anti-infectives and warrant deeper investigations. Exemplarily, we synthetically optimized the fragrance 4-(4,8-dimethyl-3,7-nonadienyl)-pyridine, a.k.a. Maritima, a pyridine derivative with marine odor. **Methods**: A new cationic *N*-cetyl-modified derivative of Maritima (dubbed Cetyl-Maritima), obtained by alkylation of Maritima, was tested for its activity against *Madurella mycetomatis* (*M. mycetomatis*) fungi, as well as against *Toxoplasma gondii* (*T. gondii*) and *Leishmania major* (*L. major*) protozoal parasites. **Results**: Cetyl-Maritima was found to be more strongly antifungal than the parent Maritima and a known antibiotic cetylpyridinium salt. Cetyl-Maritima also showed a similar activity against *T. gondii* parasites and, most notably, exhibited sub-micromolar activity against *L. major* amastigotes. **Conclusions**: The considerable antileishmanial activity of Cetyl-Maritima might lead to the development of a new potent and cost-effective drug candidate for the therapy of leishmaniasis and other infectious diseases caused by kinetoplastid parasites.

## 1. Introduction

Neglected tropical diseases (NTDs) are human infections of diverse origin, e.g., caused by viruses, bacteria, fungi, protozoa, and worms. They affect more than one billion mainly impoverished people in under-developed tropical and sub-tropical countries where proper therapies for patients are not available because of economic, infrastructural, and medical reasons [1,2]. A roadmap, recently developed by the WHO, outlines the eradication of endemic NTDs by 2030, with the costly development of new anti-infective drugs remaining a major obstacle, though [3,4]. NTD-related problems and flaws are especially eminent for mycetoma patients who suffer from one of the most neglected diseases [5,6]. People infected with eumycetoma (a fungal infection mainly caused by grain-forming *Madurella* and *Falciformispora* species) are treated with a combination of itraconazole and surgery. Patients are typically treated for six months with itraconazole before the lesion is surgically removed. During the first six months of treatment no reduction in lesion size or a decrease in (1,3)-β-D-glucan levels is noted. Only after surgery, (1,3)-β-D-glucan levels drop [7]. Under the conditions of a clinical trial, cure rates of >70% were obtained for itraconazole, and the newly validated drug fosravuconazole. However, in real-life situations, cure rates of less than 40% are obtained [8,9,10]. Thus, the search for new and more efficient antifungals for the therapy of eumycetoma is ongoing.

In contrast to the relatively low eumycetoma case numbers, leishmaniasis is an NTD caused by kinetoplastid *Leishmania* parasites and affects millions of people [11]. The disease is classified as cutaneous leishmaniasis (CL, the most abundant manifestation with up to 1 million cases), mucocutaneous leishmaniasis (MCL), and visceral leishmaniasis (VL) [12]. However, therapy options are also limited. They are mainly based on toxic medications such as pentavalent antimonials, or on expensive natural polyene drugs such as liposomal amphotericin B, which was originally developed as an antimycotic. In addition, the amidine pentamidine, the hexadecyl phosphate miltefosine, and azole drugs are applied for leishmaniasis therapy. Resistance formation and parasite species-dependent response discrepancies pose considerable medical problems [13,14]. For instance, the causative agents of Old World cutaneous leishmaniasis, *L. major* and *L. tropica*, exhibited different sensitivities to antimonials [15]. In order to eradicate leishmaniasis, the development of innovative and potent antileishmanials is necessary, including inhibitors of new drug targets and immunotherapies [16].

Other globally occurring protozoal parasite infections such as toxoplasmosis, where apicomplexan *Toxoplasma gondii* parasites are the causative agents, are a great risk for vulnerable people with suppressed immune systems who can include neonates, organ transplant patients, people suffering from HIV, etc. [17,18,19]. The current clinical arsenal of antitoxoplasmal drugs comprises sulfonamides (sulfadiazine), the dihydrofolate reductase (DHFR) inhibitors pyrimethamine and trimethoprim, and the quinone atovaquone [20]. Organ-damaging adverse effects of these drugs and the emergence of drug-resistant isolates requires the identification of new antitoxoplasmals [21,22].

**Figure 1:**
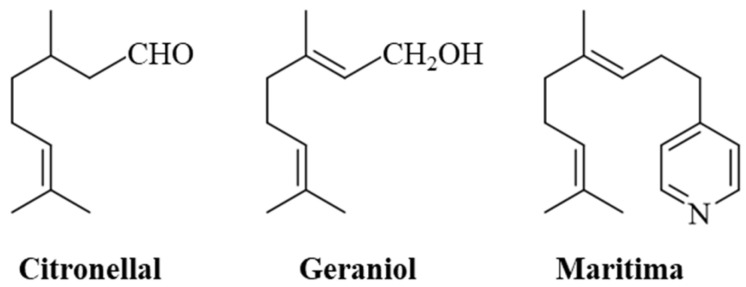
Structures of the natural fragrances citronellal and geraniol, as well as of Maritima, a semi-synthetic isoprenoid-substituted pyridine, which was used as a reference and starting compound in this study.

Fragrances are a suitable and promising source for the development of new anti-infectives. The simple natural fragrance citronellal, a monoterpene aldehyde found in *Cymbopogon* plants, showed antimicrobial, antiviral, and antiparasitic activities (Figure 1) [23,24]. In particular, citronellal exhibited antifungal activity against voriconazole-resistant *Candida albicans* strains [25]. The essential oil of *Cymbopogon winterianus* (a.k.a. Java citronella), whose major ingredients are the structurally related monoterpenes geraniol (Figure 1), citronellal, and citronellol, exhibited antiparasitic activities against *Leishmania* and *Trypanosoma* parasites [26]. Generally, natural fragrance terpenes are an important class of organic molecules. The semi-synthetic pyridine derivative Maritima, 4-(4,8-dimethyl-3,7-nonadienyl)-pyridine, has a characteristic and pleasant marine seashore odor (Figure 1) [27]. In addition, Maritima is a safe fragrance with an acceptable toxicology profile which renders this compound suitable for drug development [28].

The molecular structure of Maritima combines a pyridine ring with an isoprenoid chain. Both components of the Maritima molecule are of pharmacological relevance in their own right, and the anti-infective potential of simple monoterpenes was outlined above. The role of pyridine-based drug candidates was recently reviewed [29]. Indeed, several pyridine-based antibiotics are known [30]. The pyridine ring of Maritima seems to be amenable to chemical modifications such as *N*-alkylation or metal coordination reactions in order to obtain derivatives with new biological properties. Notably, the structurally simple *N*-hexadecyl (*N*-cetyl) substituted pyridinium compounds cetylpyridinium chloride (CPC) or bromide (CPBr) are well-established over-the-counter antimicrobials applied in topical personal care products such as mouthwashes [31,32]. Various (η^6^-arene)Ru(II) complexes of bioactive pyridines were also disclosed [33]. Thus, we prepared two new Maritima derivatives (a cetylpyridinium analog and a Ru complex) by using straightforward chemical reactions and tested their anti-infective activities against a human pathogenic fungus (*Madurella mycetomatis*) and two different protozoal parasite species (apicomplexan *T. gondii* and kinetoplastid *L. major*).

## 2. Results

### 2.1. Chemistry

**Scheme 1:**
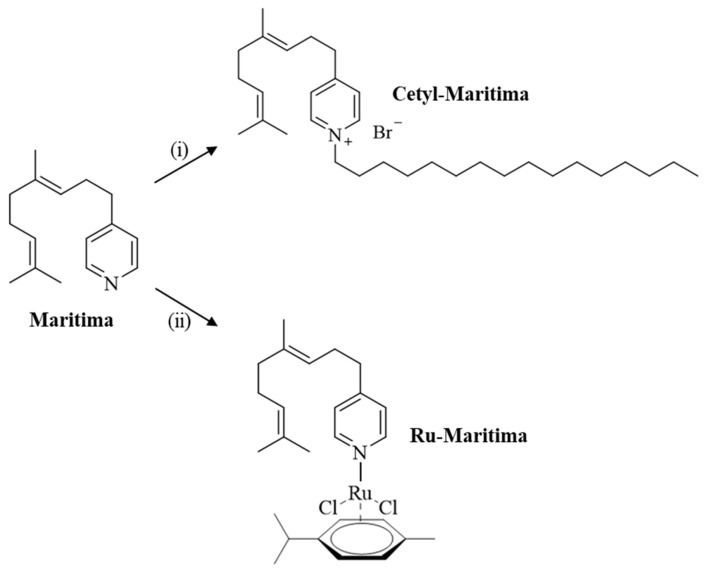
Reagents and conditions: (i) hexadecyl bromide, EtOH, reflux, 16 h, 44%; (ii) [Ru(η^6^-*p*-cymene)Cl_2_]_2_ (0.5 equiv.), CH_2_Cl_2_, 3 h, r.t., 32%.

The commercial fragrance Maritima was obtained from International Flavors & Fragrances (IFF)/Azelis and applied as a starting compound for the synthesis of the new derivatives Cetyl-Maritima and Ru-Maritima (Scheme 1). The alkylation of Maritima by reaction with hexadecyl bromide led to the hexadecylpyridinium bromide salt Cetyl-Maritima. Treatment of Maritima with 0.5 equivalents of [Ru(η^6^-*p*-cymene)Cl_2_]_2_ provided the corresponding ruthenium complex Ru-Maritima. The new compounds were analyzed by using IR, NMR, and HRMS. The obtained ^1^H and ^13^C NMR spectra exhibited the characteristic hexadecyl and *p*-cymene signals of the new Maritima derivatives (Figures S1–S4). The HRMS spectrum of Cetyl-Maritima showed the molecule peak (without bromide anion), while a chlorido ligand is missing in the spectrum of Ru-Maritima (Figures S5 and S6). Fragments missing a chlorido ligand in HRMS spectra are not without precedent and were already described in previous articles about analogous (η^6^-arene)dichloridoRu(II) complexes [34,35].

### 2.2. Antifungal Activity

The antifungal activity of Cetyl-Maritima and Ru-Maritima against *M. mycetomatis* was initially investigated according to a previously published procedure (Table 1) [36]. Their activities were compared with activities of their precursor compound Maritima, and of the known antifungals cetylpyridinium bromide (CPBr) and itraconazole, which served as positive controls. The metabolic activity of *M. mycetomatis* was suppressed by all test compounds at a high concentration of 100 μM. In particular, Ru-Maritima was highly active against *M. mycetomatis* with a negative metabolic activity percentage value. However, only Cetyl-Maritima and CPBr were strongly active at 25 μM, while fungal cells treated with Maritima still displayed 54% of metabolic activity, and cells treated with Ru-Maritima even showed enhanced metabolism (155%) at this concentration. Thus, only IC_50_ values of the most active derivatives Cetyl-Maritima and CPBr were determined. Notably, Cetyl-Maritima (IC_50_ = 8.2 μM) was approximately twice as potent as CPBr (IC_50_ = 16.0 μM). Both compounds were less active than the approved antifungal drug itraconazole.

**Figure 2:**
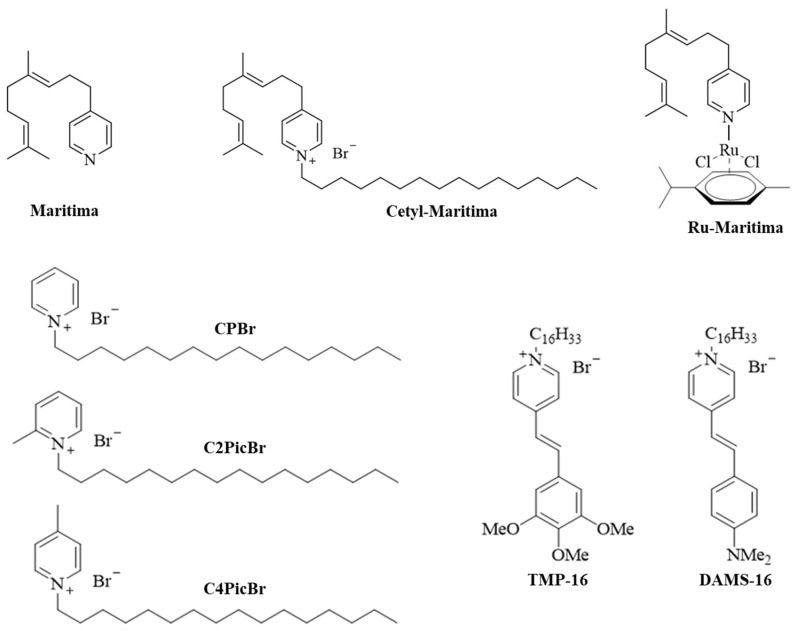
Structures of the test compounds used in the antifungal study with *M. mycetomatis*.

To assess the potential of Cetyl-Maritima as an antifungal agent, further known *N*-cetylpyridinium derivatives were tested in the *M. mycetomatis* model. These include two *N*-cetylpicolinium derivatives (C4PicBr and C2PicBr) and two *N*-cetylstilbazolium derivatives (TMP-16 and DAMS-16). However, these cetylpyridinium compounds were also less active (IC_50_ = 16.0–18.2 μM) against *M. mycetomatis* than Cetyl-Maritima, which underscores the notable antifungal activity of the new Maritima derivative. The structures of the test compounds used for antifungal evaluation are shown in Figure 2.

### 2.3. Antiparasitic Activity

Since Cetyl-Maritima showed considerable antifungal activity, it was selected for further antiparasitic evaluations. For instance, the activity of Cetyl-Maritima against *T. gondii* parasites was evaluated and compared with its toxicity to Vero kidney cells (Table 2). The starting compound Maritima, as well as the approved toxoplasmosis drug atovaquone (ATO in Table 2), were used as control compounds [37]. Maritima was inactive against *T. gondii* and Vero cells even at very high concentrations, but its derivative Cetyl-Maritima exhibited considerable activity against *T. gondii* parasites (IC_50_ = 8.6 μM) comparable with its antifungal activity against *M. mycetomatis*. While Cetyl-Maritima was distinctly less active against *T. gondii* than atovaquone, it was slightly less toxic than atovaquone to Vero cells.

Next, the activity of Cetyl-Maritima against *L. major* promastigotes and amastigotes was investigated and compared with its toxicity to macrophages (Table 3). Maritima and the approved leishmaniasis drug amphotericin B (AmB in Table 3) were applied as controls. Maritima was inactive again. However, Cetyl-Maritima exhibited high activities both against promastigotes (IC_50_ = 1.5 μM) and against amastigotes (IC_50_ = 0.6 μM), and thus *L. major* parasites were much more sensitive to Cetyl-Maritima than *T. gondii* and *M. mycetomatis* cells. Notably, Cetyl-Maritima was distinctly more active against intramacrophageal amastigotes than against promastigotes and less toxic to macrophages (IC_50_ = 4.3 μM, selectivity index/SI = 7.2). In comparison to Vero kidney cells, this SI value is even more promising (SI = 19). The positive control drug amphotericin B was only slightly more active against *L. major* than Cetyl-Maritima, and slightly less toxic to macrophages.

### 2.4. Toxicity Profile Prediction for Cetyl-Maritima

The toxicity profile prediction for Cetyl-Maritima was performed using the ProTox 3.0 platform, which calculates oral toxicities (from class 1 = fatal if swallowed to class 6 = non-toxic, and expressed as LD_50_ values), as well as the probabilities of activities and inactivities against several targets, pathways, and mechanisms associated with drug toxicity (Table S1) [38]. The predicted toxicities of Cetyl-Maritima (without bromide anion) were compared with those of Maritima and cetylpyridinium (as in CPC and CPBr without chloride/bromide anion) obtained by the same method/program (Tables S2 and S3). All three compounds were classified as oral toxicity class 4 compounds, i.e., they were predicted to be harmful if swallowed (300 mg/kg < LD_50_ < 2000 mg/kg). Maritima had the lowest predicted oral toxicity (LD_50_ = 1750 mg/kg) followed by cetylpyridinium (LD_50_ = 946 mg/kg) and Cetyl-Maritima (LD_50_ = 525 mg/kg), which might be correlated with the calculated octanol/water partition coefficient (logP) of these compounds since more toxic compounds displayed higher logP values (logP = 10.08 for Cetyl-Maritima, 6.46 for cetylpyridinium, and 4.71 for Maritima, Table 4). It is notable that Cetyl-Maritima and CPBr lack H-bond acceptors and H-bond donors, yet they have distinctly more rotatable bonds than Maritima (Table 4). The topological polar surface area (TPSA) of Cetyl-Maritima and CPBr was the same (3.88 Å^2^) and smaller than the TPSA of Maritima (12.89 Å^2^). The molecular refractivity of the cationic Cetyl-Maritima and CPBr was higher (Table 4).

**Figure 3:**
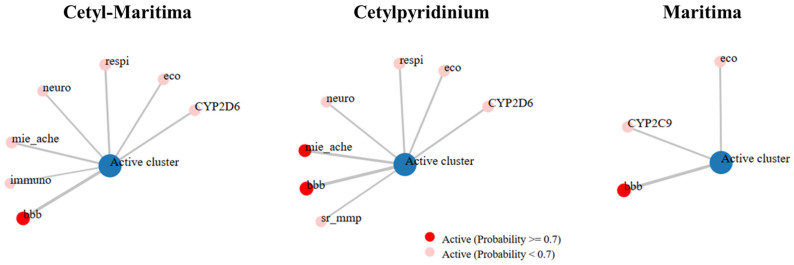
Activity clusters of the compounds Cetyl-Maritima, cetylpyridinium (as in CPC and CPBr), and Maritima. bbb = blood–brain barrier, immuno = immunotoxicity, mie_ache = molecular initiating events acetylcholinesterase, neuro = neurotoxicity, respi = respiratory toxicity, eco = ecotoxicity, sr_mmp = stress response pathway mitochondrial membrane potential, CYP = cytochrome P450.

Cetyl-Maritima is likely to cross the blood–brain barrier, which was also predicted for Maritima and cetylpyridinium (with probabilities of >0.9). For all three compounds, ecotoxicity was also predicted, albeit with lower probability (<0.7). But in total, the toxicity profile of Cetyl-Maritima was more similar to the profile of cetylpyridinium than to the profile of Maritima. The latter was predicted to be inactive against more relevant targets and mechanisms than both pyridinium compounds (Tables S1–S3). In contrast to Maritima, Cetyl-Maritima and cetylpyridinium showed certain probabilities of neurotoxicity (0.58–0.59) and respiratory toxicity (0.64–0.66). In addition, acetylcholinesterase was predicted to be a target for cetylpyridinium (0.82) and with lower probability for Cetyl-Maritima (0.67). Both compounds differed in terms of immunotoxicity (only for Cetyl-Maritima) and effects on mitochondrial membrane potentials as stress response (only for cetylpyridinium). Two metabolic targets were found with low probabilities (<0.7), CYP2C9 for Maritima and CYP2D6 for Cetyl-Maritima and cetylpyridinium. The predicted activity clusters of the compounds are shown in Figure 3.

Importantly, Cetyl-Maritima was predicted not to be hepatotoxic, nephrotoxic, cardiotoxic, carcinogenic, mutagenic, clinically toxic, or nutritionally toxic (Table S1). In addition, no interference with hormone receptors was predicted for this compound (i.e., inactive status for androgen receptor, estrogen receptor, peroxisome proliferator-activated receptor, and thyroid hormone receptor, Table S1).

## 3. Discussion

**Figure 4:**
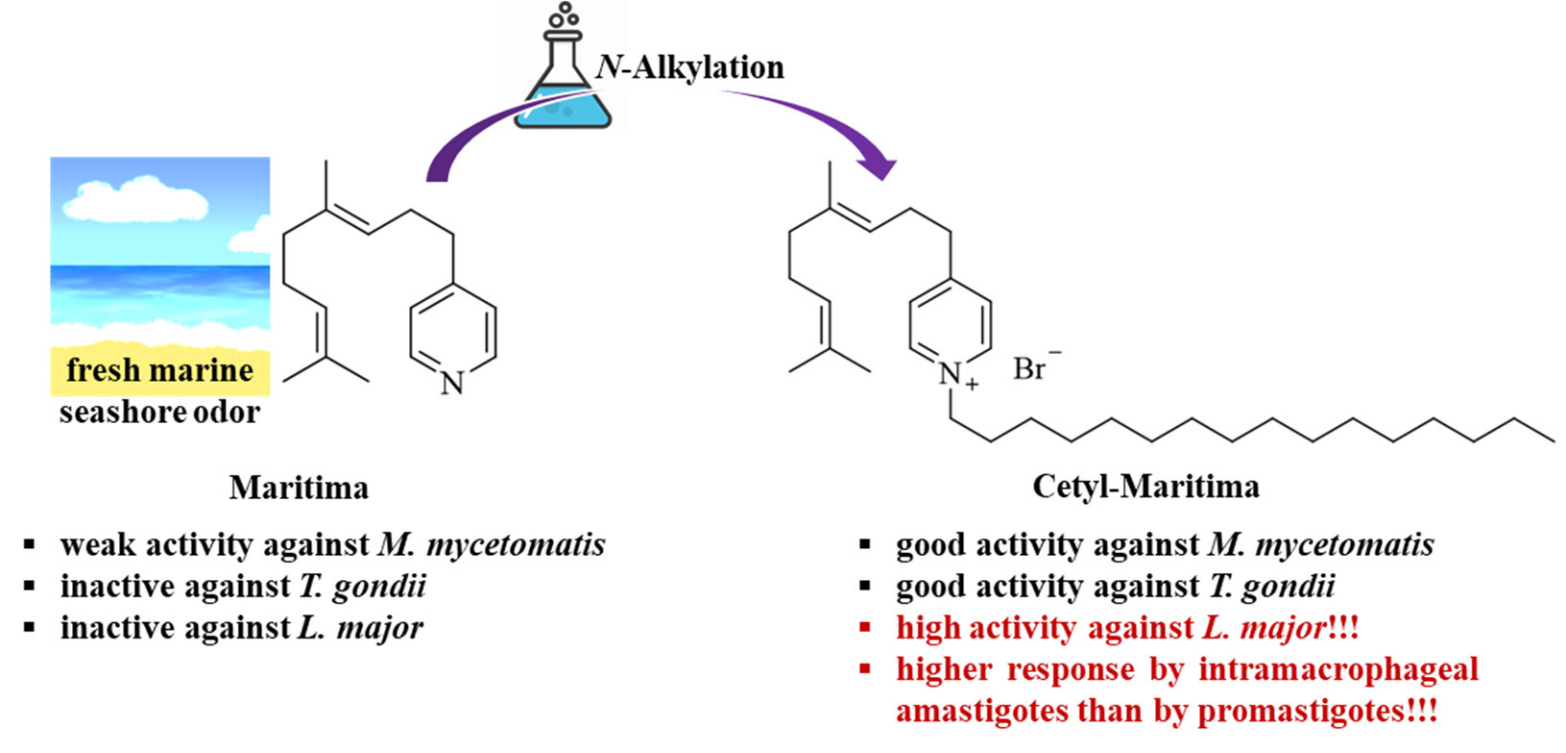
Maritima, Cetyl-Maritima, and their biological activities disclosed in this study. The odoriphore Maritima was used as starting compound for the alkylation reaction furnishing Cetyl-Maritima. Maritima was only weakly active against *M. mycetomatis* fungi and inactive against *T. gondii* and *L. major* parasites. *N*-Alkylation to Cetyl-Maritima led to significantly increased activities against *M. mycetomatis*, *T. gondii*, and *L. major*. Notably, Cetyl-Maritima was highly active against *L. major* parasites, especially against intramacrophageal amastigotes.

The pharmaceutic properties of plant-derived fragrances are well documented [39]. In order to obtain reasonable drug candidates structurally based on fragrances, the chemical modification of established fragrance molecules can be a promising strategy. We have recently shown that compounds based on the natural fragrance and spice cinnamaldehyde can become potent antitoxoplasmals through simple modifications [40]. In addition, the cinnamon oil CIN-102 exhibited in vitro and in vivo activity against *M. mycetomatis* [41]. In the current study, we successfully applied this strategy for the non-toxic commercial marine/seashore odor Maritima which is an isoprenoid-substituted pyridine derivative. The attachment of a fatty cetyl/hexadecyl moiety at the pyridine ring of Maritima, which was accomplished in one reaction step, led to a cationic *N*-hexadecylpyridinium compound, Cetyl-Maritima, with remarkable antifungal and antiparasitic properties (Figure 4).

Cetyl-Maritima turned out to be more active against *M. mycetomatis* fungi than Maritima and a known antifungal cetylpyridinium salt. In contrast to Cetyl-Maritima, the new ruthenium complex Ru-Maritima was inactive at low concentrations. Thus, *N*-alkylation of the pyridine ring of Maritima appears to be more promising in terms of the design of new Maritima-derived drugs when compared with ruthenation. It is conceivable that Cetyl-Maritima, as a mono-cationic quaternary ammonium compound (QAC), has the same mechanisms of action as the known antifungal QAC CPC used in mouthwash products [42]. The cationic pyridinium moiety of CPC interacts with anionic phosphates of cell membranes while the hexadecyl chain intercalates into the lipid bilayer of the membrane leading to its disorganization followed by leakage of cytoplasmic contents (e.g., K^+^ and pentoses already at low CPC concentrations in treated yeasts) [31]. It should be noted that the cell wall-damaging polyene macrolide amphotericin B exhibited considerable in vivo activity and was applied as positive control drug in experiments using *Galleria mellonella* larvae infected with *M. mycetomatis*, which underlines the potential of the fungal cell wall as a mycetoma drug target [43]. Resistance to CPC and other QACs was reported, and it remains to be elucidated how far known CPC-related mechanisms of resistance such as altered membrane protein profile, increased cell membrane/surface hydrophobicity, and drug efflux pumps are also relevant for Cetyl-Maritima [31]. The positively charged *N*-alkylpyridinium fragment seems to be required for activity because Maritima was distinctly less antifungal than Cetyl-Maritima, which is in line with the growing importance of QACs as antifungal agents [44]. Cationic peptides were also recently described as promising antifungal drug candidates, especially in combination with other drugs such as fluconazole or *Bidens pilosa* plant extract [45,46,47]. The discovery that Cetyl-Maritima was twice as active as CPBr against *M. mycetomatis* provides a hint at certain antifungal properties of the 4-isoprenoid substituent of Cetyl-Maritima. Additionally, *N*-hexadecyl-picolinium bromides with IC_50_ values of 16.1 μM were also less active against *M. mycetomatis* MM55 than Cetyl-Maritima (see also MycetOS website mentioned in the Data Availability Statement). Thus, a mere 4-methyl substituent did not increase the anti-mycotic activity of CPBr. *N*-Cetylstilbazolium derivatives were also less antifungal. The reason for the isoprenoid-related activity boost remains to be elucidated and might be based on further mechanisms and pathways where isoprenoid moieties play a role, possibly as competitive inhibitors. For instance, the ergosterol biosynthesis via the isoprenoid/mevalonate pathway is a well-established and specific antifungal drug target that can be addressed by using CYP51 inhibitory azole drugs [48]. Bifonazole was described as a dual CYP51/HMG-CoA-reductase inhibitor that interferes both with ergosterol synthesis and with the preceding mevalonate pathway [49,50]. Such dual targeting strategies appear to be very promising, and there are also potent CYP51 binders that can inhibit other relevant targets such as histone deacetylases and squalene epoxidase [48]. It is unlikely that Cetyl-Maritima acts as a CYP51 inhibitor because *N*-alkylpyridinium compounds cannot coordinate the catalytic heme iron of the CYP51 active site as azole drugs do [51]. But since the efficacy of azoles in eumycetoma patients is limited, new drugs are sought, and the non-azole compound Cetyl-Maritima can be considered as a suitable starting point for the design of more potent antifungals, which might be combined with azoles to achieve stronger antimycotic effects and/or improved in vivo activities [52]. It is uncertain if shorter *N*-alkyl chains will lead to higher antifungal activities, since picolinium analogs with *N*-dodecyl and *N*-tetradecyl chains displayed lower activities than *N*-hexadecyl compounds [53].

The activity of Cetyl-Maritima against protozoal parasites also revealed interesting results. The compound showed an activity against apicomplexan *T. gondii* parasites which was comparable with its activity against *M. mycetomatis* fungi (IC_50_ ca. 8 μM). In contrast, Maritima was completely inactive against *T. gondii*, while it showed some activity against *M. mycetomatis* at a concentration of 25 μM as mentioned above. In addition, Cetyl-Maritima was less toxic to renal Vero cells than atovaquone, however, its selectivity was low. Nevertheless, the reduced toxicity to Vero cells is remarkable since no nephrotoxicity was predicted for Cetyl-Maritima according to a calculation using the ProTox 3.0 platform (Table S1). How far Cetyl-Maritima can serve as a starting point for the development of an antitoxoplasmal drug remains to be shown. Both activity and selectivity need to be improved to achieve this aim. The predicted high probability of blood–brain barrier crossing for Cetyl-Maritima might be considered for future studies and drug optimizations since *T. gondii* brain cysts (cerebral toxoplasmosis) need to be tackled by new antitoxoplasmals [54]. If the predicted high blood–brain barrier crossing of Cetyl-Maritima can be verified by laboratory experiments, it might also be of relevance for other brain-infecting diseases such as human African trypanosomiasis (a.k.a. sleeping sickness, caused by various *Trypanosoma brucei* species), naegleriasis/primary amoebic meningoencephalitis (caused by the brain-eating amoeba *Naegleria fowleri*), and cryptococcal meningitis (caused by fungal *Cryptococcus* species) [55,56,57]. Notably, hydrazinated geraniol derivatives recently revealed high activities against *T. brucei rhodesiense* parasites, while extracts from *Nigella sativa* and *Thymus* sp. (which are rich in bioactive monoterpenes such as thymoquinone/TQ and thymol) were active against *T. gondii* brain cysts [58,59].

Despite the considerable activities of Cetyl-Maritima against *M. mycetomatis* fungi and *T. gondii* parasites, its antileishmanial activity is the most promising outcome of this study. Kinetoplastid *L. major* promastigotes and amastigotes turned out to be distinctly more sensitive to Cetyl-Maritima treatment than *T. gondii* and *M. mycetomatis*. In addition, Cetyl-Maritima was more active against intramacrophageal amastigotes than against promastigotes, while being distinctly less toxic to macrophage cells. Intracellular amastigotes are an attractive model because they provide information about the performance of the tested drug within host cells, which can be very useful for the development of the investigated antileishmanial drug candidate [60,61]. Approved antileishmanials including amphotericin B are very active against amastigotes, which was confirmed in our tests with *L. major* parasites [62,63]. Since Cetyl-Maritima was only slightly less active against amastigotes than amphotericin B, advanced antileishmanial testing including in vivo models appears to be promising for this new cetylpyridinium derivative. The evaluation of Cetyl-Maritima in combination with other clinically applied antileishmanials might reveal synergy effects leading to a reduction of dosage and side-effects. In addition, *Leishmania* species different from *L. major* might be evaluated for their sensitivity to Cetyl-Maritima treatment since differences in activity between *Leishmania* species were described for other drugs before [15]. Likewise, its effects on other kinetoplastid parasites such as *Trypanosoma cruzi* and *Trypanosoma brucei* should be investigated in future studies.

It appears plausible to find drug sensitivity discrepancies between fungi and protozoals because of their stark evolutionary and phylogenetic differences. But the reduced antifungal activity of Cetyl-Maritima, when compared with its antileishmanial properties, differed from the data reported for amphotericin B, which appeared to be equally active against *L. major* and *M. mycetomatis* (i.e., median inhibitory concentration/MIC and IC_50_ values between 0.25 and 2.0 μM) [64]. Instead, the activities of Cetyl-Maritima against *M. mycetomatis* fungi and protozoal *T. gondii* parasites were comparable, albeit much lower than that against *L. major*. Thus, *L. major* has certain properties (which are different from *M. mycetomatis* and *T. gondii*) that render this species especially sensitive to Cetyl-Maritima. A comparison with the only orally applicable antileishmanial drug miltefosine might be relevant in this regard [65]. The structure of the phospholipid miltefosine consists of a cetyl/hexadecyl moiety, too. In contrast to amphotericin B, which primarily binds ergosterol to damage fungal cell walls by the formation of pores, miltefosine is a pleiotropic drug targeting lipid metabolism, mitochondrial function, Ca^2+^ homeostasis, programmed cell death, and host immunity [66]. Notably, miltefosine and CPC have effects on Hsp90 in common. The anti-amoebic activity of CPC against *Entamoeba histolytica* parasites was associated with EhHsp90 inhibition by interaction with the N-terminal ATP-binding domain [67]. But in contrast to CPC, miltefosine displayed characteristics of a C-terminal Hsp90 inhibitor in human cells [68]. Nevertheless, Hsp90 might be a possible target of Cetyl-Maritima since leishmanial Hsp90 is a vital factor in the development and survival of *Leishmania* parasites that can be targeted by common Hsp90 inhibitors such as 17-AAG [69,70,71]. The known water-soluble Hsp90 inhibitor 17-DMAG cured mice infected with *L. braziliensis* based on its excellent antiparasitic and anti-inflammatory properties [72]. Hsp90 proteins of *T. gondii* and pathogenic fungi also play an increasing role in the development of new drugs against these microorganisms [73,74]. However, it is possible that structural differences between leishmanial Hsp90 and the Hsp90 proteins of other species can lead to individual responses to Hsp90 inhibitors, as was shown in a study using human and leishmanial Hsp90 [75].

In addition, cationic compounds (e.g., delocalized lipophilic compounds) are known for their mitochondria-affecting properties which is also a promising therapeutic strategy to tackle *Leishmania* parasites [76,77,78]. The quinoid monoterpene TQ was described as a mitochondria-damaging agent in cancer cells, too [79]. 17-DMAG and its natural precursor 17-AAG are also quinone derivatives. Thus, it is not surprising that TQ displayed considerable activity against *L. major* and other *Leishmania* parasites (*L. donovani*, *L. tropica*, *L. infantum*); however, promastigotes were more sensitive to TQ than amastigotes [80,81,82]. Nonetheless, the natural product class of terpenes (mono-, sesqui-, and diterpenes) is rich in promising leishmanicidal derivatives, which suggests that the isoprenoid substituent of Cetyl-Maritima also contributes to its antileishmanial activity [26,83].

The predicted toxicity profile of Cetyl-Maritima is promising and very similar to the profile of the known drug CPC. The observed SI values of Cetyl-Maritima in terms of anti-amastigote activity are considerable, in particular, in comparison with Vero kidney cells (SI = 19). For the identification of new antiparasitic hit compounds, SI values above 10 are already relevant and a good starting point for drug optimizations [84]. Such optimizations aiming at improved activity and selectivity can include chemical modifications of the Cetyl-Maritima compound on the one hand, as well as sophisticated formulations on the other hand. All three structural fragments of the Cetyl-Maritima molecule, i.e., the hexadecyl chain, the isoprenoid substituent, and the pyridine ring, can be chosen for chemical modifications and fine-tuning to improve the biological properties of the compound. In terms of formulations, liposomes are well-established carriers for amphotericin B, but liposomal amphotericin B is also expensive and thus often unavailable for patients suffering from leishmaniasis [85]. Nanocarriers based on chitosan conjugated with iron oxide nanoparticles enhanced the antifungal activity of CPC and might also be considered for Cetyl-Maritima [86]. Moreover, anionic amphotericin B combined with the cetylpyridinium cation formed an ionic liquid leading to a significant increase (3.2-fold) of the antifungal activity of amphotericin B [87]. Antibiotic formulations based on CPC were especially active against biofilms [88,89,90]. Thus, Cetyl-Maritima can be considered as a formulation system in its own right for anti-infective drugs such as amphotericin B, which would broaden the scope of this promising new fragrance-derived drug candidate.

## 4. Materials and Methods

### 4.1. Chemistry

Starting compounds were purchased from Merck (Darmstadt, Germany), Alfa Aesar (Karlsruhe, Germany), and TCI (Zwijndrecht, Belgium). The fragrance Maritima, 4-(4,8-dimethyl-3,7-nonadienyl)-pyridine, was obtained from International Flavors & Fragrances (IFF, New York, NY, USA) via its European distributor Azelis. Cetylpyridinium bromide (CPBr) was prepared from pyridine and 16-bromohexadecane in hot EtOH according to a procedure in the literature [91]. The cetylpicolinium bromides C4PicBr and C2PicBr and the cetylstilbazolium derivatives TMP-16 and DAMS-16 were prepared and published before [53,92,93]. The analytical machines used for the analysis of the new compounds (i.e., NMR, IR, and HR-MS machines) were described previously [35,37].

#### 4.1.1. 1-Hexadecyl-4-(4,8-dimethyl-3,7-nonadienyl)-pyridinium Bromide (Cetyl-Maritima)

Maritima (573 mg, 2.5 mmol) and 16-bromohexadecane (900 mg, 3.0 mmol) were dissolved in EtOH (20 mL) and stirred under reflux for 16 h. The solvent was evaporated, and the product was precipitated by addition of *n*-hexane (50 mL). The solvent was discarded, and the precipitate was dried in vacuum. Yield: 590 mg (1.11 mmol, 44%); amber gum; *ν*_max_ (ATR)/cm^−1^: 3412, 2915, 2850, 1639, 1569, 1516, 1471, 1376, 1173, 1108, 983, 839, 717, 668; ^1^H NMR (300 MHz, CDCl_3_): δ 0.85 (3 H, t, *J* = 6.7 Hz), 1.2–1.4 (26 H, m), 1.5–1.6 (6 H, m), 1.66 (3 H, s), 1.9–2.1 (6 H, m), 2.3–2.5 (2 H, m), 2.8–3.0 (2 H, m), 4.93 (2 H, t, *J* = 7.4 Hz), 5.0–5.1 (2 H, m), 7.7–7.8 (2 H, m), 9.2–9.3 (2 H, m); ^13^C NMR (75 MHz, CDCl_3_): δ 14.1, 16.2, 17.7, 22.6, 25.7, 26.0, 26.6, 27.8, 29.1, 29.3, 29.5, 29.6, 29.7, 31.9, 34.5, 36.0, 39.5, 61.3, 110.4, 120.7, 121.7, 123.6, 123.7, 123.8, 127.9, 131.7, 136.6, 138.5, 144.2, 162.6; HR-MS (ESI, *m*/*z*) for C_32_H_56_N [M^+^] calcd. 454.44073, found 454.43952.

#### 4.1.2. Dichlorido(η^6^-*p*-cymene)[4-(4,8-dimethyl-3,7-nonadienyl)-pyridine]ruthenium(II) (Ru-Maritima)

Maritima (52 mg, 0.228 mmol) was dissolved in CH_2_Cl_2_ (5 mL), and [Ru(η^6^-*p*-cymene)Cl_2_]_2_ (70 mg, 0.114 mmol) was added. The reaction mixture was stirred at room temperature for 3 h. *n*-Hexane (50 mL) was added, and the formed precipitate was collected, washed with *n*-hexane, and dried in vacuum. Yield: 40 mg (0.074 mmol, 32%); amber gum; *ν*_max_ (ATR)/cm^−1^: 3059, 2964, 2916, 2874, 1614, 1499, 1448, 1427, 1378, 1358, 1323, 1224, 1159, 1090, 1067, 1035, 1005, 985, 922, 869, 826, 802, 665; ^1^H NMR (300 MHz, CDCl_3_): δ 1.28 (6 H, d, *J* = 7.0 Hz), 1.55 (3 H, s), 1.58 (3 H, s), 1.67 (3 H, s), 1.9–2.1 (7 H, m), 2.2–2.3 (2 H, m), 2.6–2.7 (2 H, m), 2.9–3.0 (1 H, m), 5.0–5.1 (2 H, m), 5.18 (2 H, d, *J* = 6.0 Hz), 5.41 (2 H, d, *J* = 6.0 Hz), 7.0–7.1 (2 H, m), 8.8–8.9 (2 H, m); ^13^C NMR (75 MHz, CDCl_3_): δ 16.1, 17.7, 18.2, 22.3, 25.7, 26.7, 28.4, 30.6, 35.1, 39.6, 82.1, 82.8, 97.0, 103.4, 120.4, 122.1, 123.9, 124.0, 124.7, 124.8, 131.6, 137.1, 153.7, 154.2; HR-MS (ESI, *m*/*z*) for C_26_H_37_N^37^Cl^102^Ru [M^+^ − Cl] calcd. 502.16284, found 502.16399.

### 4.2. Madurella mycetomatis Cells Drug Testing Assay

The *M. mycetomatis* MM55 isolate obtained from a Sudanese patient and identified and maintained in the Erasmus Medical Centre, Rotterdam, The Netherlands, was applied for the investigation of the in vitro susceptibility of the test compounds as reported previously [94]. In short, the fungal isolate was cultured on Sabouraud agar for 2 weeks at 37 °C, after which the mycelium was transferred in colorless RPMI 1640 medium supplemented with L-glutamine (0.3 g/L; Capricorn-Scientific, Ebsdorfergrund, Germany), morpholinepropanesulfonic acid (MOPS, 20 mM, Sigma, St. Louis, MO, USA), and chloramphenicol (100 mg/L, Oxoid, Basingstroke, UK). The mycelium was sonicated for 10 s at 10 μm with a probe sonicator (Soniprep, Beun de Ronde, Abcoude, The Netherlands) to obtain a hyphal suspension, after which the hyphal suspension was cultured for another week at 37 °C. The suspension was then collected by centrifugation (5 min, 3400 rpm) and washed with PBS followed by the addition of fresh RPMI 1640 medium, after which the mycelia was again sonicated for 10 s at 10 μm with a probe sonicator. The hyphal suspension was diluted to a transmission of 70% at 660 nm (Novaspec II; Pharmacia Biotech, Uppsala, Sweden). Then, 100 μL of the suspension was added to each well of a 96-wells plate (Costar 3799, Fisher Scientific, Breda, The Netherlands) that contained 1 μL of each compound sample. Compounds were initially evaluated at concentrations of 100 μM and 25 μM. In case of growth inhibition at 25 μM, the IC_50_ values of these compounds were determined. For this reason, drug concentrations ranging from 0.09 μM to 25 μM were used and plotted (see also raw data in Table S4).

For the final metabolic activity evaluation, a viability dye named MTS, 3-(4,5-dimethylthiazol-2-yl)-5-(3-carboxymethoxyphenyl)-2-(4-sulfophenyl)-2*H*-tetrazolium salt, was applied as described in a previous study [64]. The following formula was used:

$$Percentage\;metabolic\;activity=\left(\frac{{Absorbance\;test}_{490nm}-{Absorbance\;NC}_{490nm}}{{Absorbance\;GC}_{490nm}-{Absorbance\;NC}_{490nm}}\right)*100$$

The growth control (GC) refers to the fungal isolate only and indicates 100% metabolic activity. The negative control (NC) is medium only and 0% metabolic activity was noted.

### 4.3. Toxoplasma gondii Cells Drug Testing Assay

Cells of the Vero cell line (ATCC^®^ CCL81™, Manassas, VA, USA) were used to cultivate *T. gondii* RH strain tachyzoites. The effects of test compounds on *T. gondii* growth were investigated according to previously published methods [37]. Vero cells were maintained in complete RPMI 1640 medium supplemented with 10% heat-inactivated FBS, under a humidified atmosphere containing 5% CO_2_ at 37 °C. The cells were seeded in 96-well plates at a density of 5 × 10^3^ cells/well (see also Figure S7) in 200 μL of RPMI 1640 medium and incubated at 37 °C with 5% CO_2_ for 24 h. Following incubation, the medium was discarded, and cells were washed with PBS. Subsequently, RPMI 1640 medium containing 2% FBS and *T. gondii* tachyzoites was added at a ratio of 5 parasite cells to 1 Vero cell. After 5 h incubation at 37 °C and 5% CO_2_, cells were washed with PBS and processed as outlined below.

Control: RPMI 1640 medium with 0.8% DMSO.

Experimental: RPMI 1640 medium containing test compounds (dissolved in DMSO) at concentrations of 90, 30, 10, 3.3, 1.1, 0.37, and 0.12 μg/mL. Atovaquone (ATO) was used as positive control.

Cells were incubated at 37 °C and 5% CO_2_ for 72 h, washed with PBS, fixed in 10% formalin, and stained with 1% toluidine blue. The infection index, defined as the number of infected cells out of 200 examined cells, was determined using an inverted photomicroscope.

The percentage of inhibition was calculated using the following equation:

Inhibition (%) = [(I Control − I Experimental)/I Control] × 100

where “I Control” is the infection index of untreated cells, and “I Experimental” is the infection index of cells treated with test compounds. The effects of the compounds on parasite growth were expressed as IC_50_ values, representing the concentration required to achieve 50% inhibition, from three independent experiments [37].

### 4.4. Leishmania major Drug Testing Assays

Promastigotes of *L. major* were isolated from a Saudi patient in February 2016 and cultured at 26 °C in Schneider’s Drosophila medium (Invitrogen, Carlsbad, CA, USA) supplemented with 10% heat-inactivated fetal bovine serum (FBS, Invitrogen, USA) and antibiotics. Cultures were maintained in tissue culture flasks with weekly transfers. The promastigotes were cryopreserved in liquid N_2_ at a concentration of 3 × 10^6^ parasites/mL. Virulent *L. major* parasites were maintained by serial passage in BALB/c mice through hind footpad injections of 1 × 10^6^ stationary-phase promastigotes, and amastigotes were isolated after 7 weeks. Isolated amastigotes were converted to promastigotes by culturing at 26 °C in Schneider’s medium with antibiotics and 10% FBS. Amastigote-derived promastigotes, with fewer than five in vitro passages, were used for infection experiments.

Male and female BALB/c mice were obtained from the Pharmaceutical College, King Saud University, Saudi Arabia, and housed in specific pathogen-free facilities. Animal handling followed ethical guidelines set by the Committee of Research Ethics, Deanship of Scientific Research, Qassim University (permission number 20-03-20).

*L. major* promastigotes in the logarithmic phase were cultured in phenol red-free RPMI 1640 medium (Invitrogen, USA) with 10% FBS and plated in 96-well plates at a concentration of 1 × 10^6^ cells/mL (200 μL/well) after counting with a hemocytometer. Test compounds were added at final concentrations of 90, 30, 10, 3.3, 1.1, 0.37, and 0.12 μg/mL (Table S5). Negative controls contained 0.8% DMSO without test compounds, while positive controls included decreasing concentrations of amphotericin B (AmB) (90, 30, 10, 3.3, 1.1, 0.37, and 0.12 μg/mL) as a reference compound. After 72 h of incubation at 26 °C, parasite viability was assessed via the MTT colorimetric assay. The resulting formazan was solubilized with a detergent solution, and absorbance was measured using an ELISA reader at 570 nm. IC_50_ values were determined from three independent experiments.

For amastigote activity assays, peritoneal macrophages were collected from BALB/c mice (7 weeks old) by aspiration. Cells (5 × 10^4^ cells/well) were seeded into 96-well plates (see also Figure S7) with phenol red-free RPMI 1640 medium containing 10% FBS and incubated at 37 °C with 5% CO_2_ for 4 h to allow adhesion. After discarding the medium and washing with PBS, 200 μL of *L. major* promastigotes in RPMI 1640 medium (10 promastigotes per macrophage) were added. Plates were incubated at 37 °C with 5% CO_2_ for 24 h to promote macrophage infection and amastigote differentiation. Unattached promastigotes were removed by washing with PBS (three times), and fresh phenol red-free RPMI 1640 medium containing test compounds (90, 30, 10, 3.3, 1.1, 0.37, and 0.12 μg/mL) was added. Cultures were incubated at 37 °C in 5% CO_2_ for 72 h. Negative controls contained 0.8% DMSO, while positive controls included AmB at the same concentrations. After incubation, cells were fixed and Giemsa-stained, and the percentage of infected macrophages was determined microscopically. IC_50_ values were calculated from three independent experiments. The isolation of parasites and macrophages was performed strictly following the rules and guidelines of the responsible committee of research ethics, Deanship of Scientific Research, Qassim University, Kingdom of Saudi Arabia (permission number 20-03-20) [37].

### 4.5. Vero Cell and Macrophage Cytotoxicity

MTT assays were performed to assess the cytotoxicity of the test compounds. Vero cells and isolated macrophages were seeded in 96-well plates at a density of 5 × 10^3^ cells per well in 200 μL of RPMI 1640 medium with 10% FBS and incubated at 37 °C with 5% CO_2_ for 24 h. After incubation, cells were washed with PBS and treated with test compounds at varying concentrations (90, 30, 10, 3.3, 1.1, 0.37, and 0.12 μg/mL) in 10% FBS medium for 72 h. Negative control wells contained cells treated with medium in 2% FBS without test compounds (see also raw data in Table S5). After treatment, the supernatant was removed, and 50 μL of RPMI 1640 medium containing 14 μL of MTT solution (5 mg/mL) was added. The cells were incubated for an additional 4 h. Following incubation, the supernatant was discarded, and 200 μL of DMSO was added to dissolve the formazan crystals. The absorbance was measured at 540 nm using a FLUOstar OPTIMA spectrophotometer (BMG LABTECH, Ortenberg, Germany). Cytotoxicity was quantified as IC_50_ values, representing the concentration that caused a 50% reduction in cell viability. IC_50_ values were determined from three independent experiments [37].

### 4.6. Toxicity Profile Prediction

The toxicity profiles of Cetyl-Maritima (without bromide anion), Maritima, and cetylpyridinium (without anion) were predicted using the ProTox 3.0 platform, https://tox.charite.de/protox3/index.php?site=home# (accessed on 7 January 2025) [38].

## 5. Conclusions

The identification of Cetyl-Maritima, a new anti-infective *N*-cetyl derivative of the commercially available fragrance Maritima, is of importance for various reasons. The one-step synthesis of the compound is simple and cost-effective. It is more strongly antifungal than CPBr (cetylpyridinium bromide) in *M. mycetomatis*, the causative agent of eumycetoma, and highly active against *L. major* amastigotes. The observed selectivity for *Leishmania* parasites warrants a more in-depth investigation of this compound in kinetoplastid parasites, which might also include in vivo experiments. The mechanisms of action of Cetyl-Maritima remain to be identified but are likely not different from the reported antifungal and antiprotozoal mechanisms of cetylpyridinium salts such as CPC (cetylpyridinium chloride). Nevertheless, this work shows that fragrances can be a valuable source of improved anti-infectives by making simple modifications. Future studies will show if Cetyl-Maritima can reach clinical stages of drug testing, especially for the therapy of leishmaniasis.

## Figures and Tables

**Table 1:** Antifungal activities of test compounds expressed as metabolic activity of treated *M. mycetomatis* fungal strain MM55 (as percentages ± SD and IC_50_ values). CPBr and itraconazole were used as positive controls.

Compounds	100 μM ^1^	25 μM ^1^	IC_50_ [μM]
Maritima	13.8 ± 33.8	54.3 ± 7.5	-
Cetyl-Maritima	13.1 ± 2.0	12.2 ± 6.9	8.2
Ru-Maritima	−6.6 ± 12.2	155.7 ± 22.2	-
CPBr	4.0 ± 0.8	7.9 ± 1.5	16.0
C4PicBr	5.0 ± 0.8	18.4 ± 17.7	16.1
C2PicBr	4.5 ± 0.8	8.7 ± 0.8	16.1
TMP-16	19.1 ± 1.1	13.3 ± 1.5	18.2
DAMS-16	25.4 ± 4.3	56.9 ± 69.3	16.0
Itraconazole ^2^	2.34 ± 4.49	−1.04 ± 2.37	0.09

^1^ Metabolic activity (in %) upon treatment with test compounds at doses of 100 μM and 25 μM, respectively. ^2^ Values of itraconazole also present in ref. [36].

**Table 2:** Activities (expressed as IC_50_ values ± S.D) of test compounds against *T. gondi* parasites and Vero monkey kidney cells. Atovaquone (ATO) was used as a positive control.

Compounds	*T. gondii* (μM)	Vero (μM)	SI ^1^
Maritima	>314	>314	-
Cetyl-Maritima	8.6 ± 1.2	11.4 ± 1.3	1.3
ATO ^2^	0.07 ± 0.01	9.5 ± 0.8	135

^1^ SI = selectivity index (Vero/*T. gondii*). ^2^ Values also present in ref. [37].

**Table 3:** Activities (expressed as IC_50_ values ± S.D) of test compounds against *L. major* parasites (promastigotes and intramacrophageal amastigotes) and macrophages. Amphotericin B (AmB) was used as a positive control.

Compounds	Promastigotes (μM)	Amastigotes (μM)	Macrophages (μM)	SI ^1^
Maritima	>314	>314	>314	-
Cetyl-Maritima	1.5 ± 0.2	0.60 ± 0.2	4.3 ± 0.5	7.2
AmB ^2^	0.83 ± 0.1	0.47 ± 0.2	8.1 ± 0.9	17.2

^1^ SI = selectivity index (macrophages/*L. major* amastigotes). ^2^ Values also present in ref. [37].

**Table 4:** Molecular properties and oral toxicities of the test compounds Maritima, Cetyl-Maritima, and CPBr as calculated by ProTox 3.0 [38].

Compounds	Maritima	Cetyl-Maritima	CPBr
Mol. Weight (g/mol)	229.36	454.79 (without Br^−^)	304.53 (without Br^−^)
H-Bond Acceptors	1	0	0
H-Bond Donors	0	0	0
No. of Atoms/Bonds (non-H)	17	33	22
Rotatable Bonds	6	21	15
Molecular Refractivity	76.33	154.23	102.14
Topological Polar Surface Area (Å^2^)	12.89	3.88	3.88
logP ^1^	4.71	10.08	6.46
Oral Toxicity (LD_50_, mg/kg) ^2^	1750	525	946

^1^ logP = octanol/water partition coefficient. ^2^ LD_50_ = lethal dose required to kill 50%.

## Data Availability

Original data can be obtained from the authors upon reasonable request. Antifungal activities of the test compounds Maritima (MYOS441), Cetyl-Maritima, (MYOS461), Ru-Maritima (MYOS445), CPBr (MYOS460), C2PicBr (MYOS463), C4PicBr (MYOS462), TMP-16 (MYOS464), and DAMS-16 (MYOS466) against *M. mycetomatis* can also be found on the Mycetoma Open Source/MycetOS website https://github.com/OpenSourceMycetoma (accessed on 10 March 2025).

## Supplementary Materials

The following supporting information can be downloaded at: https://www.mdpi.com/article/10.3390/antibiotics14030321/s1, original NMR spectra of test compounds Cetyl-Maritima and Ru-Maritima (Figures S1–S4), original HRMS spectra of the new compounds (Figures S5 and S6), toxicity data (Tables S1–S3), and raw data of biological experiments (Tables S4 and S5, Figure S7).
