# Supplementary material for: Antiparasitic and Antifungal Activities of Cetyl-Maritima, a New N-Cetyl-Modified Maritima Derivative
Source: Antibiotics (Basel). 2025 Mar 19;14(3):321. doi: 10.3390/antibiotics14030321 (PMC11939259; doi:10.3390/antibiotics14030321)

Supplementary Materials

Antiparasitic and Antifungal Activities of Cetyl-Maritima, a New *N*-Cetyl-Modified Maritima Derivative

Ibrahim S. Al Nasr [1], Jingyi Ma [2], Tariq A. Khan [3], Waleed S. Koko [1], Imen Ben Abdelmalek [1], Rainer Schobert [4], Wendy van de Sande [2] and Bernhard Biersack [4,*]

[1] Department of Biology, College of Science, Qassim University, Buraydah 51452, Saudi Arabia; insar@qu.edu.sa (I.S.A.N.); wasyko2002@yahoo.com (W.S.K.); mm.abdulmalek@qu.edu.sa (I.B.A.)

[2] Department of Medical Microbiology and Infectious Diseases, Erasmus MC, University Medical Center Rotterdam, Dr. Molewaterplein 40, 3015 GD Rotterdam, The Netherlands; m.jingyi@erasmusmc.nl (J.M.); w.vandesande@erasmusmc.nl (W.v.d.S.)

[3] Department of Basic Health Sciences, College of Applied Medical Sciences, Qassim University, Buraydah 51452, Saudi Arabia; sirtariqayub@gmail.com

[4] Organic Chemistry Laboratory, University Bayreuth, Universitätsstrasse 30, 95440 Bayreuth, Germany; rainer.schobert@uni-bayreuth.de

* Correspondence: bernhard.biersack@yahoo.com

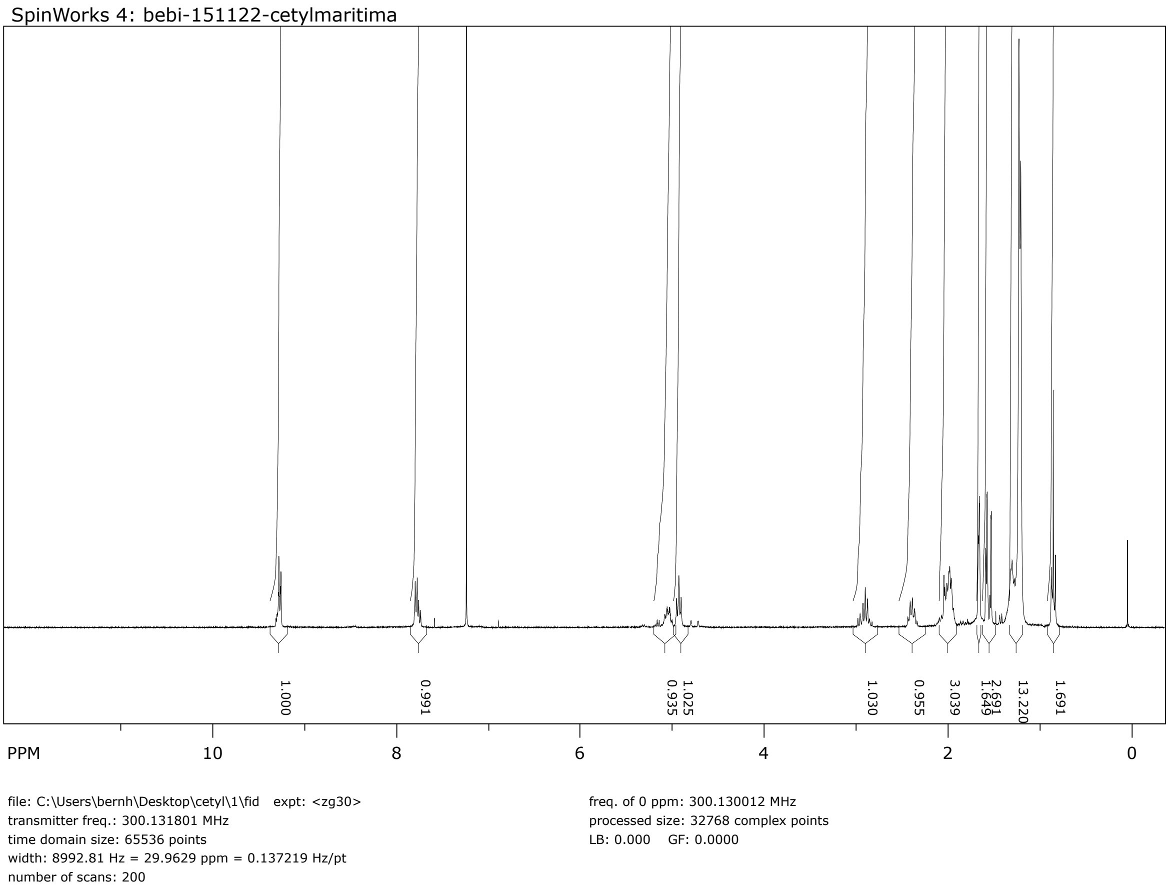

Figure S1. ^{1}H NMR spectrum of Cetyl-Maritima.

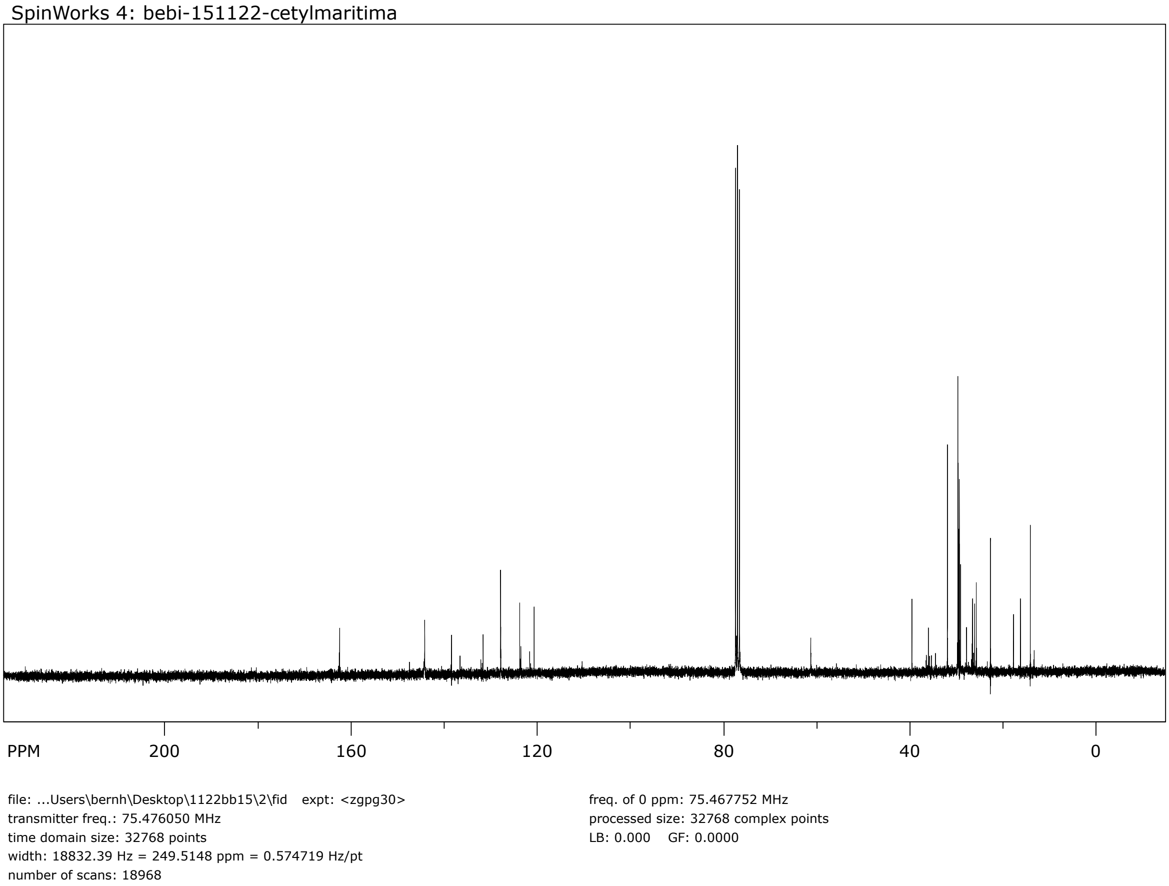

Figure S2. ^{13}C NMR spectrum of Cetyl-Maritima.

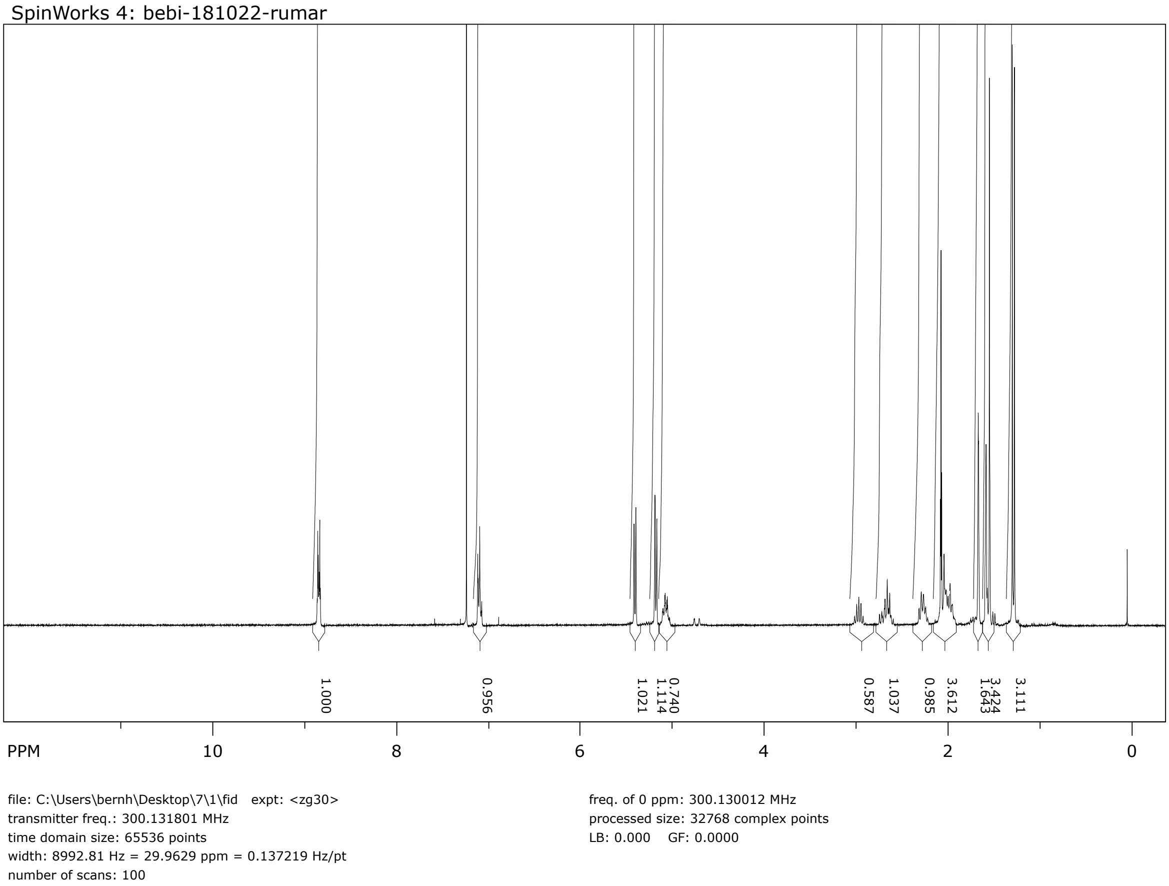

Figure S3. ^{1}H NMR spectrum of Ru-Maritima.

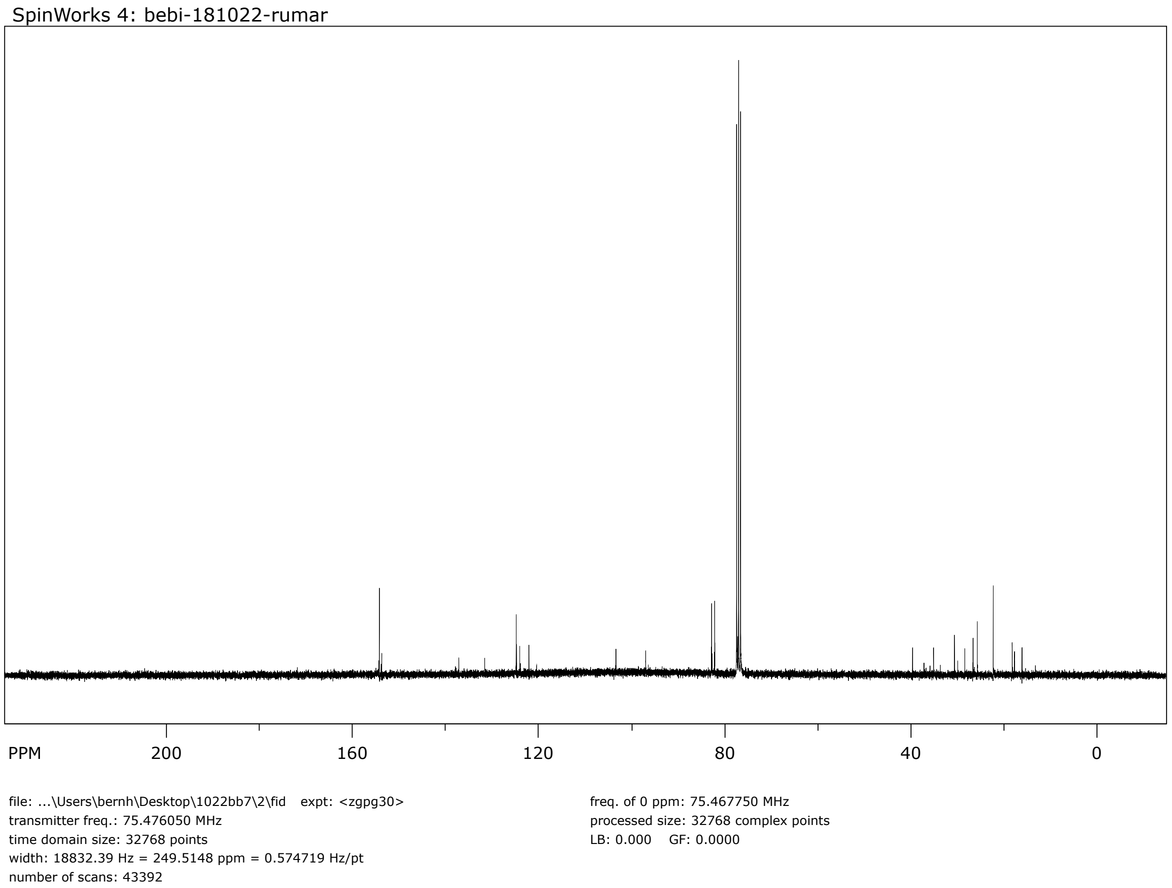

Figure S4. ^{13}C NMR spectrum of Ru-Maritima.

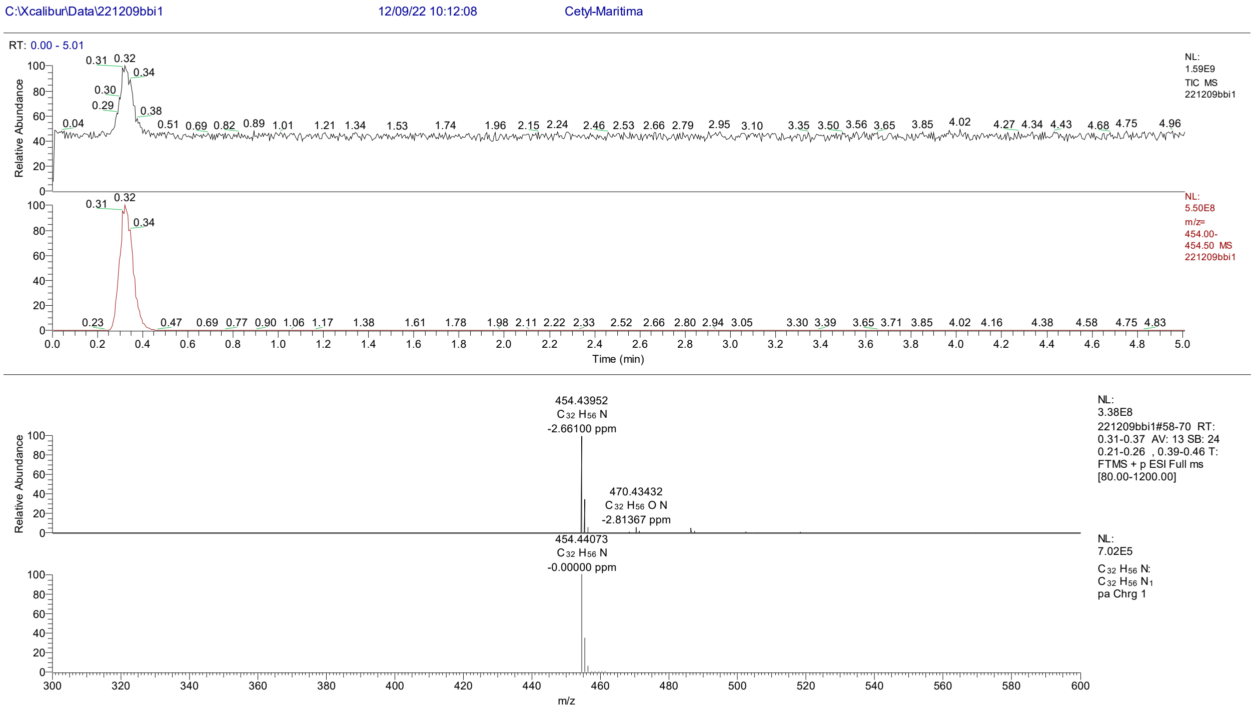

Figure S5. HRMS spectrum of Cetyl-Maritima.

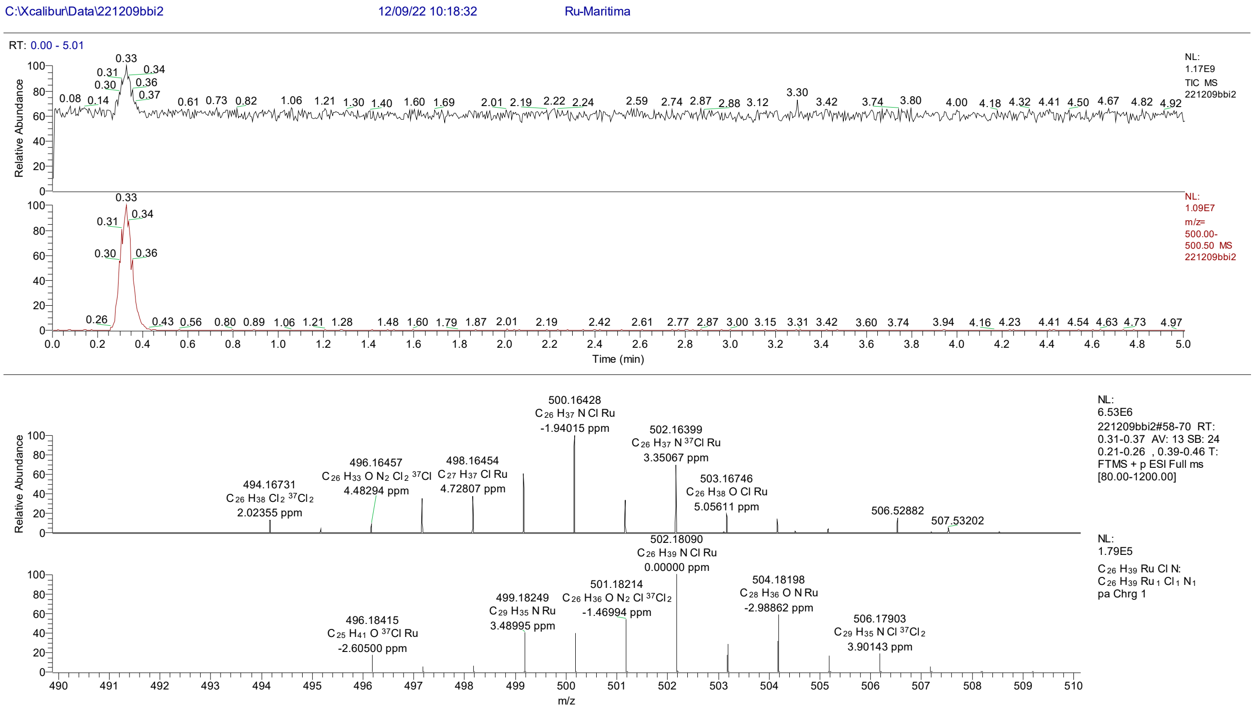

Figure S6. HRMS spectrum of Ru-Maritima.

Table S1. Predicted toxicity profile of Cetyl-Maritima.

Classification	Target	Shorthand	Prediction	Probability
Organ toxicity	Hepatotoxicity	dili	Inactive	0.85
Organ toxicity	Neurotoxicity	neuro	Active	0.58
Organ toxicity	Nephrotoxicity	nephro	Inactive	0.90
Organ toxicity	Respiratory toxicity	respi	Active	0.66
Organ toxicity	Cardiotoxicity	cardio	Inactive	0.80
Toxicity end points	Carcinogenicity	carcino	Inactive	0.73
Toxicity end points	Immunotoxicity	immuno	Active	0.50
Toxicity end points	Mutagenicity	mutagen	Inactive	0.77
Toxicity end points	Cytotoxicity	cyto	Inactive	0.75
Toxicity end points	BBB-barrier	bbb	Active	0.94
Toxicity end points	Ecotoxicity	eco	Active	0.61
Toxicity end points	Clinical toxicity	clinical	Inactive	0.76
Toxicity end points	Nutritional toxicity	nutri	Inactive	0.84
Tox21-Nuclear receptor signalling pathways	Aryl hydrocarbon Receptor (AhR)	nr_ahr	Inactive	0.96
Tox21-Nuclear receptor signalling pathways	Androgen Receptor (AR)	nr_ar	Inactive	0.99
Tox21-Nuclear receptor signalling pathways	Androgen Receptor Ligand Binding Domain (AR-LBD)	nr_ar_lbd	Inactive	0.99
Tox21-Nuclear receptor signalling pathways	Aromatase	nr_aromatase	Inactive	0.76
Tox21-Nuclear receptor signalling pathways	Estrogen Receptor Alpha (ER)	nr_er	Inactive	0.83
Tox21-Nuclear receptor signalling pathways	Estrogen Receptor Ligand Binding Domain (ER-LBD)	nr_er_lbd	Inactive	0.97
Tox21-Nuclear receptor signalling pathways	Peroxisome Proliferator Activated Receptor Gamma (PPAR-Gamma)	nr_ppar_gamma	Inactive	0.98
Tox21-Stress response pathways	Nuclear factor (erythroid-derived 2)-like 2/ antioxidant responsive element (nrf2/ARE)	sr_are	Inactive	0.89
Tox21-Stress response pathways	Heat shock factor response element (HSE)	sr_hse	Inactive	0.89
Tox21-Stress response pathways	Mitochondrial Membrane Potential (MMP)	sr_mmp	Inactive	0.51
Tox21-Stress response pathways	Phosphoprotein (Tumor Supressor) p53	sr_p53	Inactive	0.99
Tox21-Stress response pathways	ATPase family AAA domain-containing protein 5 (ATAD5)	sr_atad5	Inactive	0.99
Molecular Initiating Events	Thyroid hormone receptor alpha (THRα)	mie_thr_alpha	Inactive	0.72
Molecular Initiating Events	Thyroid hormone receptor beta (THRβ)	mie_thr_beta	Inactive	0.85
Molecular Initiating Events	Transtyretrin (TTR)	mie_ttr	Inactive	0.91
Molecular Initiating Events	Ryanodine receptor (RYR)	mie_ryr	Inactive	0.93
Molecular Initiating Events	GABA receptor (GABAR)	mie_gabar	Inactive	0.80
Molecular Initiating Events	Glutamate N-methyl-D-aspartate receptor (NMDAR)	mie_nmdar	Inactive	0.85
Molecular Initiating Events	alpha-amino-3-hydroxy-5-methyl-4-isoxazolepropionate receptor (AMPAR)	mie_ampar	Inactive	1.0
Molecular Initiating Events	Kainate receptor (KAR)	mie_kar	Inactive	1.0
Molecular Initiating Events	Achetylcholinesterase (AChE)	mie_ache	Active	0.67
Molecular Initiating Events	Constitutive androstane receptor (CAR)	mie_car	Inactive	0.99
Molecular Initiating Events	Pregnane X receptor (PXR)	mie_pxr	Inactive	0.54
Molecular Initiating Events	NADH-quinone oxidoreductase (NADHOX)	mie_nadhox	Inactive	0.73
Molecular Initiating Events	Voltage gated sodium channel (VGSC)	mie_vgsc	Inactive	0.7
Molecular Initiating Events	Na+/I- symporter (NIS)	mie_nis	Inactive	0.85
Metabolism	Cytochrome CYP1A2	CYP1A2	Inactive	0.88
Metabolism	Cytochrome CYP2C19	CYP2C19	Inactive	0.78
Metabolism	Cytochrome CYP2C9	CYP2C9	Inactive	0.56
Metabolism	Cytochrome CYP2D6	CYP2D6	Active	0.64
Metabolism	Cytochrome CYP3A4	CYP3A4	Inactive	0.88
Metabolism	Cytochrome CYP2E1	CYP2E1	Inactive	0.93

Table S2. Predicted toxicity profile of cetylpyridinium.

Classification	Target	Shorthand	Prediction	Probability
Organ toxicity	Hepatotoxicity	dili	Inactive	0.90
Organ toxicity	Neurotoxicity	neuro	Active	0.59
Organ toxicity	Nephrotoxicity	nephro	Inactive	0.91
Organ toxicity	Respiratory toxicity	respi	Active	0.64
Organ toxicity	Cardiotoxicity	cardio	Inactive	0.73
Toxicity end points	Carcinogenicity	carcino	Inactive	0.68
Toxicity end points	Immunotoxicity	immuno	Inactive	0.95
Toxicity end points	Mutagenicity	mutagen	Inactive	0.77
Toxicity end points	Cytotoxicity	cyto	Inactive	0.70
Toxicity end points	BBB-barrier	bbb	Active	0.98
Toxicity end points	Ecotoxicity	eco	Active	0.64
Toxicity end points	Clinical toxicity	clinical	Inactive	0.81
Toxicity end points	Nutritional toxicity	nutri	Inactive	0.69
Tox21-Nuclear receptor signalling pathways	Aryl hydrocarbon Receptor (AhR)	nr_ahr	Inactive	0.99
Tox21-Nuclear receptor signalling pathways	Androgen Receptor (AR)	nr_ar	Inactive	0.99
Tox21-Nuclear receptor signalling pathways	Androgen Receptor Ligand Binding Domain (AR-LBD)	nr_ar_lbd	Inactive	0.99
Tox21-Nuclear receptor signalling pathways	Aromatase	nr_aromatase	Inactive	0.61
Tox21-Nuclear receptor signalling pathways	Estrogen Receptor Alpha (ER)	nr_er	Inactive	0.97
Tox21-Nuclear receptor signalling pathways	Estrogen Receptor Ligand Binding Domain (ER-LBD)	nr_er_lbd	Inactive	0.99
Tox21-Nuclear receptor signalling pathways	Peroxisome Proliferator Activated Receptor Gamma (PPAR-Gamma)	nr_ppar_gamma	Inactive	0.99
Tox21-Stress response pathways	Nuclear factor (erythroid-derived 2)-like 2/ antioxidant responsive element (nrf2/ARE)	sr_are	Inactive	0.99
Tox21-Stress response pathways	Heat shock factor response element (HSE)	sr_hse	Inactive	0.99
Tox21-Stress response pathways	Mitochondrial Membrane Potential (MMP)	sr_mmp	Active	0.56
Tox21-Stress response pathways	Phosphoprotein (Tumor Supressor) p53	sr_p53	Inactive	0.99
Tox21-Stress response pathways	ATPase family AAA domain-containing protein 5 (ATAD5)	sr_atad5	Inactive	0.99
Molecular Initiating Events	Thyroid hormone receptor alpha (THRα)	mie_thr_alpha	Inactive	0.67
Molecular Initiating Events	Thyroid hormone receptor beta (THRβ)	mie_thr_beta	Inactive	0.84
Molecular Initiating Events	Transtyretrin (TTR)	mie_ttr	Inactive	0.93
Molecular Initiating Events	Ryanodine receptor (RYR)	mie_ryr	Inactive	0.92
Molecular Initiating Events	GABA receptor (GABAR)	mie_gabar	Inactive	0.83
Molecular Initiating Events	Glutamate N-methyl-D-aspartate receptor (NMDAR)	mie_nmdar	Inactive	0.72
Molecular Initiating Events	alpha-amino-3-hydroxy-5-methyl-4-isoxazolepropionate receptor (AMPAR)	mie_ampar	Inactive	1.0
Molecular Initiating Events	Kainate receptor (KAR)	mie_kar	Inactive	1.0
Molecular Initiating Events	Achetylcholinesterase (AChE)	mie_ache	Active	0.82
Molecular Initiating Events	Constitutive androstane receptor (CAR)	mie_car	Inactive	0.99
Molecular Initiating Events	Pregnane X receptor (PXR)	mie_pxr	Inactive	0.69
Molecular Initiating Events	NADH-quinone oxidoreductase (NADHOX)	mie_nadhox	Inactive	0.84
Molecular Initiating Events	Voltage gated sodium channel (VGSC)	mie_vgsc	Inactive	0.78
Molecular Initiating Events	Na+/I- symporter (NIS)	mie_nis	Inactive	0.95
Metabolism	Cytochrome CYP1A2	CYP1A2	Inactive	0.88
Metabolism	Cytochrome CYP2C19	CYP2C19	Inactive	0.79
Metabolism	Cytochrome CYP2C9	CYP2C9	Inactive	0.79
Metabolism	Cytochrome CYP2D6	CYP2D6	Active	0.65
Metabolism	Cytochrome CYP3A4	CYP3A4	Inactive	0.97
Metabolism	Cytochrome CYP2E1	CYP2E1	Inactive	0.92

Table S3. Predicted toxicity profile of Maritima.

Classification	Target	Shorthand	Prediction	Probability
Organ toxicity	Hepatotoxicity	dili	Inactive	0.76
Organ toxicity	Neurotoxicity	neuro	Inactive	0.60
Organ toxicity	Nephrotoxicity	nephro	Inactive	0.89
Organ toxicity	Respiratory toxicity	respi	Inactive	0.55
Organ toxicity	Cardiotoxicity	cardio	Inactive	0.84
Toxicity end points	Carcinogenicity	carcino	Inactive	0.71
Toxicity end points	Immunotoxicity	immuno	Inactive	0.98
Toxicity end points	Mutagenicity	mutagen	Inactive	0.85
Toxicity end points	Cytotoxicity	cyto	Inactive	0.81
Toxicity end points	BBB-barrier	bbb	Active	0.95
Toxicity end points	Ecotoxicity	eco	Active	0.60
Toxicity end points	Clinical toxicity	clinical	Inactive	0.67
Toxicity end points	Nutritional toxicity	nutri	Inactive	0.86
Tox21-Nuclear receptor signalling pathways	Aryl hydrocarbon Receptor (AhR)	nr_ahr	Inactive	0.96
Tox21-Nuclear receptor signalling pathways	Androgen Receptor (AR)	nr_ar	Inactive	0.98
Tox21-Nuclear receptor signalling pathways	Androgen Receptor Ligand Binding Domain (AR-LBD)	nr_ar_lbd	Inactive	0.98
Tox21-Nuclear receptor signalling pathways	Aromatase	nr_aromatase	Inactive	0.88
Tox21-Nuclear receptor signalling pathways	Estrogen Receptor Alpha (ER)	nr_er	Inactive	0.58
Tox21-Nuclear receptor signalling pathways	Estrogen Receptor Ligand Binding Domain (ER-LBD)	nr_er_lbd	Inactive	0.97
Tox21-Nuclear receptor signalling pathways	Peroxisome Proliferator Activated Receptor Gamma (PPAR-Gamma)	nr_ppar_gamma	Inactive	0.99
Tox21-Stress response pathways	Nuclear factor (erythroid-derived 2)-like 2/ antioxidant responsive element (nrf2/ARE)	sr_are	Inactive	0.83
Tox21-Stress response pathways	Heat shock factor response element (HSE)	sr_hse	Inactive	0.83
Tox21-Stress response pathways	Mitochondrial Membrane Potential (MMP)	sr_mmp	Inactive	0.83
Tox21-Stress response pathways	Phosphoprotein (Tumor Supressor) p53	sr_p53	Inactive	0.99
Tox21-Stress response pathways	ATPase family AAA domain-containing protein 5 (ATAD5)	sr_atad5	Inactive	0.99
Molecular Initiating Events	Thyroid hormone receptor alpha (THRα)	mie_thr_alpha	Inactive	0.85
Molecular Initiating Events	Thyroid hormone receptor beta (THRβ)	mie_thr_beta	Inactive	0.93
Molecular Initiating Events	Transtyretrin (TTR)	mie_ttr	Inactive	0.76
Molecular Initiating Events	Ryanodine receptor (RYR)	mie_ryr	Inactive	0.93
Molecular Initiating Events	GABA receptor (GABAR)	mie_gabar	Inactive	0.75
Molecular Initiating Events	Glutamate N-methyl-D-aspartate receptor (NMDAR)	mie_nmdar	Inactive	0.86
Molecular Initiating Events	alpha-amino-3-hydroxy-5-methyl-4-isoxazolepropionate receptor (AMPAR)	mie_ampar	Inactive	0.99
Molecular Initiating Events	Kainate receptor (KAR)	mie_kar	Inactive	1.0
Molecular Initiating Events	Achetylcholinesterase (AChE)	mie_ache	Inactive	0.72
Molecular Initiating Events	Constitutive androstane receptor (CAR)	mie_car	Inactive	0.99
Molecular Initiating Events	Pregnane X receptor (PXR)	mie_pxr	Inactive	0.54
Molecular Initiating Events	NADH-quinone oxidoreductase (NADHOX)	mie_nadhox	Inactive	0.52
Molecular Initiating Events	Voltage gated sodium channel (VGSC)	mie_vgsc	Inactive	0.77
Molecular Initiating Events	Na+/I- symporter (NIS)	mie_nis	Inactive	0.86
Metabolism	Cytochrome CYP1A2	CYP1A2	Inactive	0.81
Metabolism	Cytochrome CYP2C19	CYP2C19	Inactive	0.70
Metabolism	Cytochrome CYP2C9	CYP2C9	Active	0.62
Metabolism	Cytochrome CYP2D6	CYP2D6	Inactive	0.54
Metabolism	Cytochrome CYP3A4	CYP3A4	Inactive	0.79
Metabolism	Cytochrome CYP2E1	CYP2E1	Inactive	0.89

Table S4. Raw data of antifungal experiments with *Madurella mycetomatis*.

	name	Mw		100uM-1	100uM-2	100uM-3	average	stdev	25uM-1	25uM-2	25uM-3	average	stdev
MYOS_00460_04_01	Cetylpyridinium	384,45		4,882917	3,431022	3,667201	3,993713	0,779074	9,561605	7,558971	6,532203	7,884259	1,540675
MYOS_00461_04_01	Cetyl-Maritima	534,71		13,59048	10,81129	14,78341	13,06173	2,038161	20,08865	8,059328	8,365803	12,17126	6,858375
MYOS_00462_04_01	Cetyl-4-picolinium	398,47		5,922625	4,431737	4,584002	4,979455	0,82035	38,80341	7,308792	9,168004	18,42673	17,67118
MYOS_00463_04_01	Cetyl-2-picolinium	398,47		5,402771	4,056469	4,125602	4,528281	0,758119	9,561605	8,559685	7,907403	8,676231	0,833237
MYOS_00464_04_01	TMP-16	576,66		20,08865	17,94139	19,25281	19,09428	1,082375	11,77099	13,31308	14,78341	13,28916	1,506353
MYOS_00466_04_01	DAMS-16	529,65		29,96588	24,94639	21,31561	25,40929	4,343677	18,91898	136,9014	14,89801	56,90612	69,30708
MYOS_00441_04_01	Maritima	286,48		-6,9	-4,5	52,9	13,8	33,8	50,3	49,7	62,9	54,3	7,5
MYOS_00445_04_01	Ru-Maritima	537,58		0	1	-20,7	-6,6	12,2	152,3	184,8	142,5	155,7	22,2

		25	6,25	1,56	0,39	0,09	**IC50**		25	6,25	1,56	0,39	0,09	**IC50**	**leanIC50**
MYOS_00460_04_01		7,728091	79,15666	8,463385	205,3121	132,8331	**13,75**		8,865504	124,1026	171,3277	136,4134	8,159147	**18,3**	**16,025**
MYOS_00461_04_01		11,19448	42,07683	79,57683	68,23229	97,95918	**5,25**		9,168228	64,56682	87,97751	111,6909	110,3791	**11,17**	**8,21**
MYOS_00462_04_01		10,45918	98,37935	8,358343	172,1188	88,71549	**16,51**		8,865504	91,20657	148,7242	146,6052	90,60112	**15,64**	**16,075**
MYOS_00463_04_01		7,202881	74,11465	139,3457	99,7449	86,08944	**13**		8,360963	141,8625	147,3115	75,26308	81,72121	**19,15**	**16,075**
MYOS_00464_04_01		12,66507	83,2533	88,5054	96,4886	95,64826	**15,1**		42,56883	79,60213	147,3115	92,92201	119,7636	**21,24**	**18,17**
MYOS_00466_04_01		14,97599	88,40036	98,69448	95,85834	162,455	**16**		69,91495	80,91394	129,3499	74,96036	8,159147	**/**	**16**
MYOS_00441_04_01															
MYOS_00445_04_01															

Table S5. Plate design and raw data of toxicity experiments with Vero cells, macrophages, and *L. major* promastigotes.

Design of plates													
				**Maritima**	**Cetyl-Maritima**				**Ctrl +ve**	**Negative Control (0.8 % DMSO)**			Conc of cpd
**A**	Hydration	Hydration	Hydration	Hydration	Hydration	Hydration	Hydration	Hydration	Hydration	Hydration	Hydration	Hydration	
**B**	Hydration	Hydration									Hydration	Hydration	90.00
**C**	Hydration	Hydration									Hydration	Hydration	30.00
**D**	Hydration	Hydration									Hydration	Hydration	10.00
**E**	Hydration	Hydration									Hydration	Hydration	3.33
**F**	Hydration	Hydration									Hydration	Hydration	1.11
**G**	Hydration	Hydration									Hydration	Hydration	0.37
**H**	Hydration	Hydration	Hydration	Hydration	Hydration	Hydration	Hydration	Hydration	Hydration	Hydration	Hydration	Hydration	

Vero cells

0.043	0.042	0.042	0.042	0.04	0.042	0.039	0.039	0.036	0.043	0.043	0.042
0.043	0.042	0.302	0.323	0.133	0.122	0.274	0.312	0.126	0.563	0.037	0.039
0.043	0.042	0.333	0.553	0.115	0.112	0.278	0.702	0.111	0.669	0.043	0.043
0.038	0.055	0.329	0.728	0.119	0.174	0.27	0.732	0.131	0.758	0.04	0.043
0.04	0.038	0.498	0.718	0.151	0.439	0.466	0.784	0.143	0.785	0.034	0.044
0.042	0.037	0.589	0.718	0.167	0.643	0.574	0.756	0.156	0.779	0.07	0.043
0.043	0.039	0.922	0.935	0.522	0.945	0.895	0.935	0.495	0.944	0.036	0.045
0.042	0.042	0.041	0.042	0.042	0.052	0.067	0.049	0.042	0.044	0.044	0.041
0.043	0.042	0.039	0.044	0.014	0.043	0.039	0.038	0.04	0.041	0.045	0.045
0.044	0.042	0.362	0.371	0.152	0.14	0.315	0.358	0.144	0.647	0.037	0.039
0.043	0.042	0.394	0.635	0.132	0.128	0.319	0.807	0.127	0.769	0.044	0.041
0.038	0.055	0.346	0.837	0.136	0.2	0.31	0.841	0.15	0.871	0.05	0.041
0.044	0.038	0.488	0.825	0.173	0.504	0.535	0.901	0.164	0.902	0.041	0.044

0.042	0.037	0.569	0.825	0.192	0.739	0.662	0.869	0.179	0.895	0.06	0.043
0.041	0.039	0.576	1.075	0.601	1.086	1.029	1.075	0.569	1.086	0.055	0.043
0.039	0.042	0.038	0.039	0.041	0.044	0.057	0.044	0.042	0.044	0.48	0.044

0.039	0.043	0.043	0.044	0.043	0.045	0.041	0.042	0.042	0.052	0.052	0.049
0.043	0.042	0.331	0.31	0.114	0.121	0.383	0.302	0.143	0.568	0.067	0.042
0.043	0.039	0.314	0.49	0.106	0.131	0.288	0.714	0.121	0.569	0.042	0.039
0.038	0.037	0.356	0.721	0.112	0.166	0.298	0.714	0.128	0.758	0.043	0.045
0.04	0.038	0.478	0.688	0.135	0.4	0.452	0.765	0.138	0.743	0.044	0.039
0.042	0.042	0.563	0.708	0.149	0.546	0.513	0.77	0.151	0.869	0.067	0.035
0.043	0.055	0.899	0.884	0.392	0.846	0.789	0.788	0.441	0.886	0.049	0.046
0.042	0.042	0.041	0.037	0.07	0.034	0.04	0.036	0.042	0.044	0.042	0.043

Macrophages

0.044	0.045	0.044	0.045	0.045	0.046	0.043	0.045	0.045	0.041	0.044	0.044
0.044	0.045	0.3	0.194	0.211	0.126	0.117	0.123	0.159	0.409	0.045	0.043
0.044	0.045	0.256	0.468	0.305	0.109	0.111	0.185	0.121	0.534	0.044	0.042
0.045	0.044	0.276	0.694	0.399	0.104	0.144	0.221	0.123	0.616	0.042	0.043
0.043	0.045	0.452	0.703	0.554	0.103	0.325	0.318	0.114	0.563	0.044	0.043
0.044	0.045	0.537	0.644	0.595	0.126	0.609	0.378	0.135	0.571	0.044	0.043
0.044	0.044	0.6	0.522	0.519	0.248	0.562	0.418	0.427	0.534	0.044	0.042
0.043	0.043	0.046	0.043	0.043	0.04	0.045	0.043	0.045	0.042	0.044	0.044

0.045	0.044	0.041	0.044	0.046	0.043	0.045	0.044	0.041	0.044	0.041	0.042
0.043	0.042	0.313	0.199	0.198	0.117	0.122	0.123	0.166	0.511	0.044	0.043
0.044	0.043	0.312	0.511	0.423	0.143	0.136	0.185	0.124	0.563	0.045	0.044
0.043	0.043	0.388	0.621	0.431	0.145	0.156	0.221	0.136	0.571	0.045	0.044
0.045	0.043	0.424	0.681	0.497	0.233	0.178	0.318	0.138	0.601	0.043	0.043
0.044	0.042	0.568	0.638	0.565	0.563	0.207	0.378	0.141	0.561	0.045	0.046
0.042	0.04	0.574	0.587	0.578	0.577	0.417	0.418	0.453	0.732	0.045	0.044
0.044	0.045	0.045	0.043	0.046	0.041	0.043	0.045	0.044	0.044	0.044	0.045

0.046	0.042	0.045	0.041	0.044	0.041	0.043	0.044	0.041	0.045	0.043	0.043
0.041	0.043	0.266	0.203	0.148	0.198	0.156	0.135	0.162	0.638	0.041	0.039
0.045	0.042	0.301	0.439	0.111	0.414	0.165	0.21	0.115	0.623	0.044	0.044
0.041	0.041	0.313	0.557	0.136	0.411	0.178	0.235	0.131	0.688	0.045	0.041
0.039	0.044	0.504	0.678	0.139	0.52	0.256	0.336	0.144	0.781	0.038	0.043
0.044	0.045	0.548	0.662	0.144	0.533	0.425	0.382	0.17	0.715	0.046	0.035
0.041	0.043	0.599	0.631	0.201	0.613	0.509	0.511	0.513	0.621	0.041	0.044
0.046	0.042	0.04	0.044	0.041	0.046	0.042	0.043	0.045	0.044	0.043	0.046

Antipromastigote activities of test compounds

0.056	0.046	0.045	0.044
0.451	0.116	0.096	0.568
0.835	0.168	0.123	0.895
0.956	0.143	0.185	0.998
0.942	0.225	0.213	1.006
0.862	0.299	0.265	0.975
0.97	0.635	0.453	0.965
0.044	0.042	0.043	0.048

0.044	0.041	0.044	0.048
0.856	0.151	0.136	0.689
933	0.135	0.149	1.135
1.632	0.167	0.168	1.356
1.441	0.289	0.201	1.336
1.567	0.401	0.338	1.541
1.345	0.868	0.454	1.442
0.045	0.051	0.052	0.48

0.109	0.038	0.042	0.055
0.612	0.141	0.112	0.731
1.292	0.128	0.131	1.138
1.459	0.12	0.135	1.264
1.552	0.256	0.186	1.301
1.491	0.311	0.241	1.325
1.399	0.906	0.416	1.307
0.042	0.096	0.07	0.07

Figure S7: Microplate schemes and activities of *L. major* amastigote and *T. gondii* experiments.

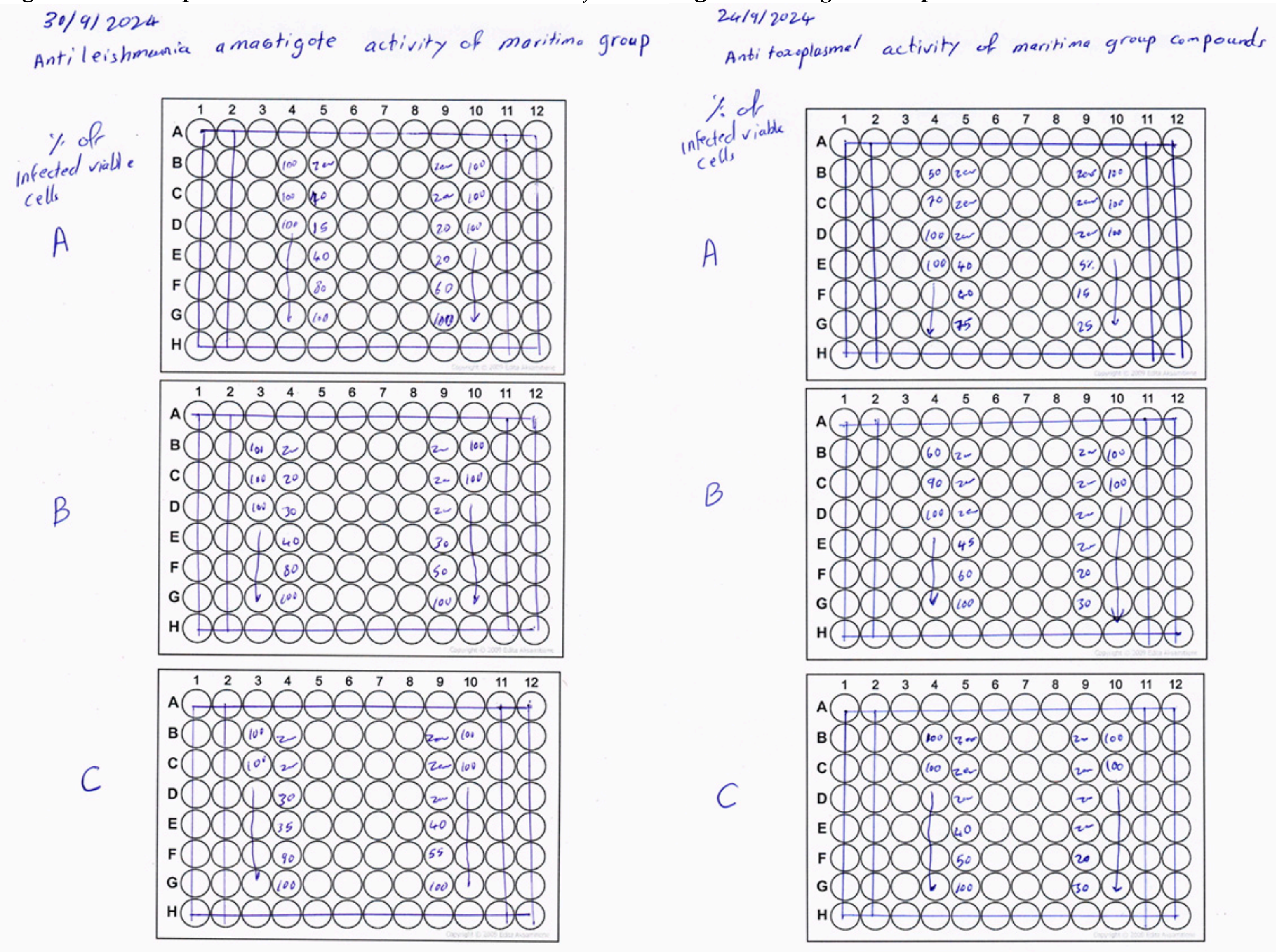

Supplement: Supplementary file 1 [file antibiotics-14-00321-s001.zip › antibiotics-3517772-supplementary.pdf]
